# Grouped semantic-feature relation extraction from texts to represent medicinal-plant property knowledge on social media

**DOI:** 10.3389/frai.2025.1579357

**Published:** 2025-08-08

**Authors:** Chaveevan Pechsiri, Intaka Piriyakul, Joseph Santhi Pechsiri

**Affiliations:** ^1^College of Innovative Technology and Engineering, Dhurakij Pundit University, Bangkok, Thailand; ^2^Faculty of business administration for society, Srinakharinwirot University, Bangkok, Thailand; ^3^Department of Forest Biomaterials and Technology, Swedish University of Agricultural Sciences, Uppsala, Sweden

**Keywords:** grouped semantic-feature relation, word co-occurrence, structural equation modeling, natural language processing (computer science), artificial intelligence

## Abstract

This research aims to extract a grouped semantic-feature relation, particularly a PlantPart-MedicinalPropertyGroup relation which is a semantic relation between an element of a plant-part concept set and a group of medicinal-property concept features of various herbs or medicinal plants, including indigenous medicinal plants, to graphically represent medicinal-plant property knowledge from documents available on pharmacy academic websites. The medicinal-plant property knowledge representation particularly benefits native users and patients seeking alternative medical therapies during pandemics, such as COVID-19, due to limited access to medicines, physicians and hospitals. Medicinal-property expressions on the documents, particularly in Thai, are often structured as event expressions conveyed through verb phrases within Elementary Discourse Units (EDUs) or simple sentences. There are three research problems in extracting the PlantPart-MedicinalPropertyGroup relations from the documents: how to identify EDU occurrences with medicinal-property concepts, how to extract medicinal-property concept features from medicinal-property concept EDU occurrences without concept annotations, and how to extract the PlantPart-MedicinalPropertyGroup relation without relation-class labeling from the documents with the high dimensional and correlated feature consideration. To address these problems, we apply a Solving-Verb Concept set primarily sourced from translated terms on HerbMed, an American Botanical Council resource, to identify a medicinal-property concept EDU. Additionally, a word co-occurrence (word-co) pattern is applied as a compound variable on the translated terms to construct a medicinal-property-concept (MPC) table. The MPC table is employed to extract the medicinal-property concept features from the medicinal-property concept EDUs through a string-matching method. We then propose using structural equation modeling to automatically extract the PlantPart-MedicinalPropertyGroup relations from the documents. Thus, the proposed approach enables the extraction of PlantPart-MedicinalPropertyGroup relations with high qualities to represent medicinal-plant property knowledge on social media.

## 1 Introduction

During a pandemic, e.g., the COVID-19 pandemic, indigenous medicinal plants from various countries were considered as herbal medicines in an alternative medical therapy (Nelson and Perrone, 2000) due to the limited availability of conventional medicines, physicians and hospitals. The majority of herbal medicine usages from indigenous medicinal plants are formed through herbal-indigenous knowledge, where indigenous knowledge is the systematic body of knowledge acquired by local people through the accumulation of experiences, informal experiments, and an intimate understanding of the environment in a given culture (Rajasekaran, 1993). A combination of indigenous medicinal plants was used synergistically in COVID-19 symptom treatment. Synergistic effects are the combined effects of at least two different medicinal plants/plant-parts with different phytochemicals or biological structures that have a greater influence than either one of them could have individually (Prasathkumar et al., 2021; Ali et al., 2022; Intharuksa et al., 2022). Regarding (Rajasekaran, 1993; Yuan et al., 2006; Itharat et al., 2021; Petrovska, 2012), the awareness of plant-part usage from medicinal plants as a natural product is a result of many years of struggles against illnesses. Additionally, the medicinal properties in various parts (i.e., roots, rhizomes, leaves, flowers, seeds, etc.) of the plant vary in contents and percentages of the plant-part phytochemicals. These phytochemicals not only protect plants from competitors, pathogens, or predators, but also protect humans and animals against certain diseases (Parihar and Balekar, 2016; Osman et al., 2020; Rabizadeh et al., 2022) that result in similar or different properties (Agidew, 2022). There are about 200 different medicinal plants in Thailand with various medicinal properties found in different plant parts, as documented by the Plant Genetic Conservation Project under the royal initiative of Her Highness Princess Maha Chakri Sirindhorn (http://www.rspg.or.th/; accessed on 18 August 2024; Singhabutra, 1992) in cooperation with the Pharmacy Division of the Department of Health, Thailand. However, it is time consuming to read all of the medicinal properties of all medicinal plants from documents to find each semantic relation which is the logical or conceptual relation expressed in a sentence or sentences in the text (Khoo and Na, 2006). The semantic relation pertinent to our research is a grouped semantic-feature relation connecting an element of a semantic-feature set to a group of related semantic features. Specifically, it involves a PlantPart-MedicinalPropertyGroup relation (called a “pp-mpG” relation) which is a semantic relation connecting a *pp*_*i*_ feature (*pp*_*i*_ ∈ PP which is a plant-part concept set) to *mpG*_*g*_—a group of related medicinal-property concept features where *mpG*_*g*_ ⊂ MPG which is a set of medicinal-property concept groups from the medicinal plants. Thus, determining the grouped semantic-feature relation, i.e., the “pp-mpG” relation, from downloaded Thai documents for graphical representation of medicinal-plant property knowledge on social media is a challenge. Moreover, Khoo and Na (2006) also stated that “the sematic relation is the cement linking up the concepts into the knowledge structures.”

**Figure 1:**
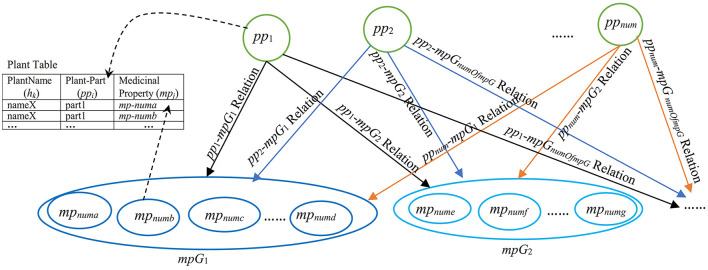
A graphical representation of the pp-mpG relations including Plant Table for finding plant names by *pp*_*i*_ and/or *mp*_*j*_ where all *pp*_1_-*mp*G_1_, *pp*_1_-*mp*G_2_, *pp*_1_-*mp*G_*numOfmpG*_, *pp*_2_-*mp*G_1_, *pp*_2_-*mp*G_2_, *pp*_2_-*mp*G_*numOfmpG*_, …, *pp*_*num*_-*mp*G_1_, *pp*_*num*_-*mp*G_2_, *pp*_*num*_-*mp*G_*numOfmpG*_ relations are the pp-*mp*G relations connecting *pp*_*i*_ (a plant-part concept feature, *i* = 1,2,..,*num*) to *mp*G_*g*_ (a group of medicinal-property concept features, *g* = 1,2,.., *numOfmpG* in MPG which is a set of medicinal-property concept groups).

Numerous previous studies (Khoo and Na, 2006; Fang et al., 2008; Choi and Lee, 2015; Behera and Mahalakshmi, 2019; Cho et al., 2020; Yoo et al., 2020; Zhang et al., 2020; Jia et al., 2022; Braik et al., 2023; Pechsiri and Piriyakul, 2016; see Table A.1) determined or computerized a relation, particularly a semantic relation as a traditional relation linking a medicinal-plant name (which is a medicinal-plant/herbal name concept) to a medicinal property (a medicinal-property/phytochemical-activity concept) for disease prevention and/or treatment from the corpus by manually or semi-automatically annotating the relation without concerning the high dimensional and correlated feature space of various medicinal properties (see Section 2). According to Salmerón-Manzano et al. (2020), it is estimated that globally there are ~350,000–1,000,000 plant species used for medicinal purposes, highlighting the vast scope of this domain. Our research observations reveal significant correlation occurrences among medicinal-property concepts that depend on various plant names or plant-part concepts; for instance, both “*antiviral*” and “*anti-inflammatory*” concepts frequently co-occur in medicinal plant names such as “*curcumin*,” “*ginger*,” and “*lemongrass*,” whilst “*antipyretic*,” “*antitussive*,” and “*expectorant*” concepts also appear together in some plant-part concepts like lemon fruit and miracle tree. These correlation patterns demonstrate the complex, interconnected nature of medicinal property concepts across different plant species, necessitating sophisticated analytical approaches to properly model these multidimensional relationships in medicinal plant research. Therefore, this research aims to automatically extract the grouped semantic-feature relation as the pp-mpG relation which occurs as a common relation (see Figure 1) between *pp*_*i*_ and *mpG*_*g*_ of various *h*_*k*_ from the Thai documents downloaded from several web sites of organizations involving in herb and medicinal plant researches, e.g., Herbal Information Center at the Faculty of Pharmacy-Mahidol University (https://medplant.mahidol.ac.th/document/inews.asp; accessed on 18 August 2024), Faculty of Pharmacy-Silpakorn University (https://pharmacy.su.ac.th/herbmed/herb/text/; accessed on 18 August 2024), and (http://www.rspg.or.th/; accessed on 18 August 2024) where:

1) *h*_*k*_ is a medicinal-plant name concept which is also used as a document file name, *k* = 1,2,.., *numOfHerbalNameConcepts*.
2) *pp*_*i*_ is a plant-part concept feature which is an element of a plant-part concept set (PP), *pp*_*i*_ ∈ PP; PP = {“*root*,” “*rhizome*,” “*leaf*,” “*flower*,” “*seed*,”...}, and *i* = 1,2,.., *numOfPlantPartConcepts* in PP; -
3) *mp*_*j*_ is a medicinal-property concept element, particularly the medicinal-action/activity concept element such as “*anti*-*inflammation*,” “*wound healing*,” “*anti*-*virus growth*,” etc., and *mp*_*j*_ ∈ MP where MP is the universal medicinal-property concept set having *j* = 1,2,.., *numOfMedicinalPropertyConcepts* in MP;
4) *mpG*_*g*_ is a group of medicinal-property concept features having some correlations among their features.
*mpG*_*g*_ = {*mp*_1_, *mp*_2_,…, *mp*_*last*−*g*_} which contains several *mp*_*j*_ features having some *mp*_*j*_ features that are correlated, and *g* = 1,2,.., *numOfmpG* in MPG;
*mpG*_*g*_ ⊂ MPG on which MPG is a set of medicinal-property concept groups from these medicinal plants; MPG = {*mpG*_1_,*mpG*_2_,..*mpG*_*numOfmpG*_};
5) *mpG*_1_ ∪ *mpG*_2_…∪ *mpG*_*numOfmpG*_ = MP; *mpG*_*g*_ and *mpG*_*q*_ are mutually exclusive for *g* ≠ *q*; and 1 ≤ *q* ≤ *numOfmpG*.

Each pp-mpG relation is involved with the high dimensional and correlated features of medicinal-property concepts.

Figure 1 presents a representation of medicinal-plant property knowledge where each pp-mpG relation as a linkage is represented by an arrow connecting a *pp*_*i*_ feature node to a *mpG*_*g*_ node, which consists of several *mp*_*j*_ features nodes with some correlations among the *mp*_*j*_ features; *i* = 1, 2, ..., *num* (*num* is *numOfPlantPartConcepts*); *numa, numb, numc, numd, nume, numf*, and *numg* are different numbers of *j*. Figure 1 also includes a Plant table (Plant Table) consisting of PlantName or *h*_*k*_, Plant-Part or *pp*_*i*_, and MedicinalProperty or *mp*_*j*_ collected from the documents. The Plant table is used to decide whether to list either the *h*_*k*_ features along with *pp*_*i*_ features with the same *mp*_*j*_ feature or vice versa. Moreover, the synergistic effect typically occurs if the particular *mpG*_*g*_ node is linked to different *pp*_*i*_ features.

Thus, the pp-mpG relations shown in Figure 1 present the medicinal-plant property knowledge for potential usage in an alternative medical therapy through social media. The medicinal-plant property knowledge representation benefits individuals and communities with limited access to medical facilities and modern treatments. The usage of mixing medicinal plants not only has a synergistic effect, but also has low cost and minimal side effects compared to certain western medicines (Behera and Mahalakshmi, 2019).

**Figure 2:**
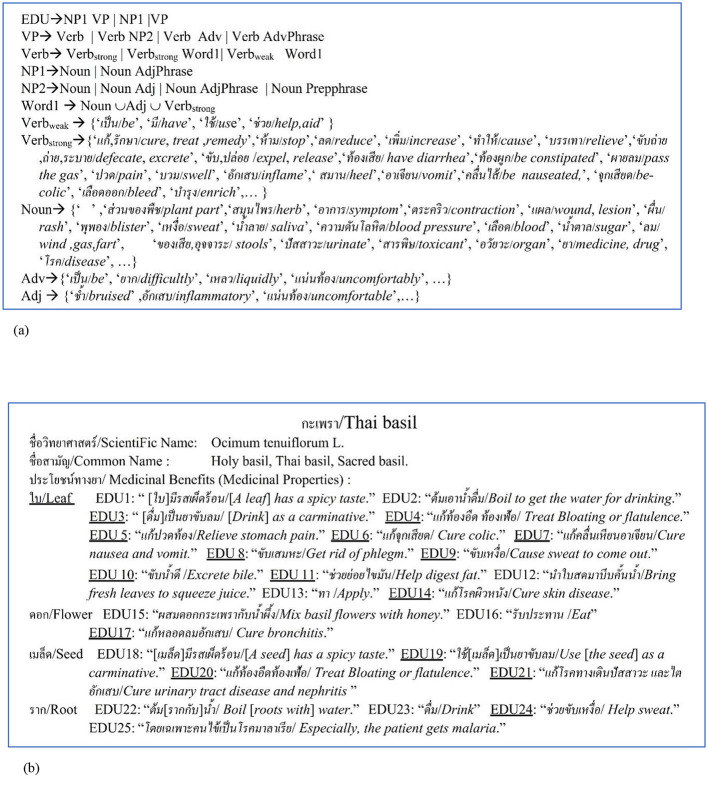
Example: a medicinal plant or herb document, e.g., “Thai basil,” based on **(a)** a general Thai linguistic expression including Thai-to-English translation by Lexitron Dictionary and WordNet where NP1 and NP2 are noun phrases; VP is a verb phrase; Verb is a verb term, Verb_strong_ is a strong-verb concept set, Verb_weak_ is a weak-verb concept set, Adv is an adverb concept set, Adj is an adjective concept set, and Noun is a noun concept set. **(b)** An explanation by several EDUs with medicinal property concepts where a [...] symbol means a word/words ellipsis.

The *mp*_*j*_ feature expression on the documents is mostly based on an event expression of a medicinal-property activity on an elementary discourse unit (EDU), which is defined as a simple sentence or a clause by Carlson et al. (2003). The event expression is explained by a verb with the event semantic (Pustejovsky, 1991) on the EDU's verb phrase wherein each EDU expression is based on a general linguistic expression, e.g., a general Thai linguistic expression (see Figure 2a) after stemming words and eliminating stop words.

In addition, the medicinal property concepts of our research rely on HerbMed (provided by the American Botanical Council; https://www.herbalgram.org/resources/terminology/; accessed on 18 August 2024) including the medical-symptom term list on the Wikipedia web site (https://en.wikipedia. https://www.org/wiki/List_of_medical_symptoms; accessed on 18 August 2024). Each medicinal property concept by HerbMed and each medical-symptom-term concept by Wikipedia is presented in English as either a noun term or a noun phrase expression. In contrast, the corresponding concepts in Thai are expressed by an EDU or an EDU's verb phrase. The noun or noun phrase representing either a medicinal property concept from HerbMed or a medical symptom concept from Wikipedia is translated into a Thai term represented by an EDUtmt—a translated EDU expression in Thai, derived from the English translation of the corresponding concept in HerbMed or Wikipedia, using Lexitron, a Thai-English machine-readable dictionary developed by NECTEC (the National Electronics and Computer Technology Center of Thailand; http://lexitron.nectec.or.th/, accessed on 20 August 2024; Trakultaweekoon et al., 2007). The reason for using the medical-symptom term list is because some medicinal-property terms do not occur in HerbMed as it only presents the most common terms of the medicinal properties of plants. The EDU_tmt_ expressions are then segmented by the Thai word segmentation tool (Sudprasert and Kawtrakul, 2003) which yields the elements, including the element concepts of the Verb_weak_, Verb_strong_, Adv, Adj, and Noun sets in Figure 2a where the element concepts are determined by the Thai-to-English translation with Lexitron including WordNet (Miller, 1995).

For example: HerbMed term, e.g., “*carminative*” is translated to the Thai language as “ขับลม/$\bar{\text{k}}$hạblm” which is an EDU_tmt_ expression. The result of the (“ขับลม/$\bar{\text{k}}$hạblm”)/EDU_tmt_ segmentation including a part of speech notification is ((“ขับ/$\bar{\text{k}}$hạb”/*expel*/Verb_strong_ “ลม/lm”/*gas*/Noun)/VP)/EDU_tmt_ which conveys the same concept as “*carminative*” (see Section 3.2).

Regarding the automatic extraction of the grouped semantic-feature relation (the pp-mpG relation) from the documents, e.g., the Thai basil document in Figure 2b, there are three main research problems.

1) How to automatically identify several EDU occurrences with medicinal-property concepts scattered throughout the downloaded documents, which are mostly semi-structure data. For example: several EDU occurrences (EDU_*r*_; *r* = 1,2,..,*numOfDocumentEDUs*) in Figure 2b have 15 different *mp*_*j*_ feature expressions based on verb phrases of EDU3-EDU11, EDU14, EDU17, EDU19-EDU21, and EDU24, where a [..] symbol means an ellipsis of a word or words inside the symbol.
2) How to extract the *mp*_*j*_ features from the medicinal-property concept EDU occurrences without annotating the concepts of the *mp*_*j*_ features on the EDU occurrences.
3) How to extract the grouped semantic-feature relation, which is the pp-mpG relation/linkage between *pp*_*i*_ and *mpG*_*g*_ involving the high dimensional and correlated feature space of the *mp*_*j*_ features.

In order to fulfill the aim of automatically extracting a grouped semantic-feature relation from the documents, this paper will conduct the following:

1) Use a Solving-Verb Concept set (SVC), {“แก้,รักษา/*cure*,*treat*,*remedy*,” “ลด/*reduce*,” “บรรเทา/*relieve*,” “เป็น/*be*+ยา/*medicine*,” “ช่วย/*help*+ขับ/*excrete*,”…}, to identify each medicinal-property concept EDU from the documents. According to the general Thai linguistic expression on Figure 2a, SVC is a verb set with the solving concept, formed by the verb term (Verb) consisting Verb_strong_, Verb_strong_ + Word1, or Verb_weak_ + Word1. This contrasts with previous research (Behera and Mahalakshmi, 2019) which used terms based on noun expressions from MeSH to identify phytochemical-property/medicinal-property sentences from PubMedCloud.
2) Apply a compound variable, i.e., a word co-occurrence (called word-co) to represent the medicinal-property term concepts and the medical-symptom term concepts from HerbMed and Wikipedia, respectively. The word-co expression relies on the following word-co pattern (called “WCPattern”) on which WCPattern is the verb-based word-co pattern to represent events on the EDU_tmt_ after stemming words and stop word removal. WCPattern is then used to construct a medicinal-property-concept (MPC) table from the translated terms of HerbMed and Wikipedia. The MPC table is used to extract *mp*_*j*_ features from medicinal-property concept EDUs by a string-matching method.
$$\begin{array}{l} WCPattern:SVC+W1+W2\;+W3 \end{array}$$
where: regarding the Verb_weak_, Verb_strong_, Adv, Adj, and Noun sets on Figure 2, W1, W2, and W3 are the problem/symptom-word concept sets and exist right after SVC in sequence where (*w*_1_ ∈ W1) ≠ (*w*_2_ ∈ W2) ≠ (*w*_3_ ∈ W3); W1, W2, and W3 = Noun∪Adj∪Adv∪Verb_strong_. If ((*v*_*strong*_ ∈ SVC) ∨ (*v*_*weak*_ + *wrd*) ∈ SVC)) + a “อาการ/*symptom*” word, then the next word right after the “อาการ/*symptom*” word is *w*_1_, followed by *w*_2_ and *w*_3_ in sequence where *v*_*strong*_ ∈ Verb_strong_, *v*_*weak*_ ∈ Verb_weak_, and *wrd* ∈ Word1. And, if *w*_1_, *w*_2_, or *w*_3_ does not exist, then *w*_1_, *w*_2_, or *w*_3_ is null, respectively. For example, “แก้/ *cure*” + “อาการ/*symptom*” + “ปวด/*pain*” + “กล้ามเนื้อ/*muscle*” = “แก้/*cure*” + “ปวด/*pain*” + “กล้ามเนื้อ/*muscle*.”
3) Propose a statistical-based approach by using structural equation modeling (SEM), a multivariate statistical analysis technique (Schumacker and Lomax, 2004) to extract the pp-mpG relation on the high dimensional and correlated *mp*_*j*_ features from the downloaded documents to represent the medicinal-plant property knowledge on social media without relation-class annotation on the documents. The neural network is more complicated than SEM and requires relation-class annotation or labeling. Generally, machine-learning techniques, except neural networks, without considering the feature correlation between either dependent variables or independent variables, are not suitable for our corpus in extracting the pp-mpG relation. Moreover, most of the traditional relation approaches to relation classification and extraction heavily rely on rule bases and machine learning techniques without concerning the correlation among features (Detroja et al., 2023). Therefore, our research approach both a proposed SEM technique and a machine-learning technique, particularly a support vector machine (SVM; Cristianini and Shawe-Taylor, 2000), to observe the quantitative and qualitative differences between the extracted relations/linkages on the correlated features in the documents by SEM and SVM techniques. According to this research, SVM determines and extracts several *pp*_*i*_-*mp*_*j*_ pairs with pp-mp relations between *pp*_*i*_ features and *mp*_*j*_ features having the same *pp*_*i*_ feature. SEM is applied to determine and extract the pp-mpG relations from the documents after applying hierarchical factor analysis, which consists of two levels: a first-order factor model and a higher-order factor model, where each level is a part of the general linear model used to reduce numerous variables/features into fewer numbers of factors without information loss (Kim and Mueller, 1978).

Moreover, the following are key terminologies used in this paper.

- EDU is an elementary discourse unit which is defined as a simple sentence or a clause.
- EDU_tmt_ is a translated EDU expression by translating the medicinal-property/medical-symptom terms on HerbMed/the Wikipedia, respectively, from English to Thai by Lexitron.
- MPC Table is Medicinal-Property-Concept Table.
- pp-mpG is a semantic relation linking an element of plant-part concept set to a group of related medicinal-property concept features.
- pp-mp is a semantic relation between a *pp*_*i*_ feature and various *mp*_*j*_ features on *pp*_*i*_-*mp*_*j*_ pairs with the same *pp*_*i*_ feature.
- SEM is Structural Equation Modeling.
- SVC is a verb set with the solving concept; an SVC element is formed by the verb term (Verb) consisting Verb_strong_, Verb_strong_ + Word1, or Verb_weak_ + Word1 on the EDU_tmt_ occurrence.
- SVM is Support Vector Machine.
- word-co is a word co-occurrence.
- WCPattern is the verb-based word-co pattern on the translated EDU expression (EDU_tmt_) of events after stemming words and stop word removal.

Our research is organized into five sections. In Section 2, related works are summarized. Our methodology shows a system framework for extracting the pp-mpG relations and pp-mp relations in Section 3. We evaluate our proposed model including discussion in Section 4. Finally, in Section 5, the conclusions are provided.

## 2 Related works

Most of the previous research studies (Fang et al., 2008; Choi and Lee, 2015; Behera and Mahalakshmi, 2019; Cho et al., 2020; Yoo et al., 2020; Zhang et al., 2020; Jia et al., 2022; Braik et al., 2023; Pechsiri and Piriyakul, 2016) attempted to determine a relation or association between the medicinal plants in an herbal category and their medicinal properties or between phytochemical activities and diseases in the documents, including medical entity recognition.

Fang et al. (2008) used TCM (Traditional Chinese Medicine) names involving natural products that included many effective chemical components from herbs, gene names, disease names, TCM ingredients, and effects from a TCMGeneDIT database to annotate the PubMed literature corpus by employing an NLP (Natural Language Processing) tool. They found various associations, including (TCM, gene), (TCM, disease), (TCM, gene, disease), (TCM, ingredient), (TCM, effect), and (gene, ingredient), by applying rule-based information extraction to extract the relations between effecters and effects from the corpus and also the transitive association based on the Swanson's ABC model. The average precision result of the associations was 0.91 at 95% minimum confidence without emphasizing the high dimensional feature space on (TCM, disease), (TCM, ingredient), and (TCM, effect).

Choi and Lee (2015) applied a rule-based text mining model to infer herb-chemical relationships from a corpus of 245 PubMed abstracts that were annotated as herb–chemical relation by three annotators. The F-measure of the rule-based model for identifying herb-chemical relationships was 0.749 by testing with the PubMed abstracts.

Behera and Mahalakshmi (2019) applied text mining techniques to biomedical literatures on PubMed Cloud to reveal information concerning the curing of disease based upon the phytochemical properties of medicinal plants. The diseases and the phytochemical properties based on noun expressions were identified from the literature by using selected terms based on the probabilistic term frequency of MeSH thesaurus in research articles from PubMed. The result of identifying an informative sentence pertaining to cure/treat disease, have side effects and prevent relationship types by a trained probabilistic classifier displayed 73% accuracy where the positions of the disease feature and the phytochemical property feature occurred anywhere in the sentence.

Cho et al. (2020) sought medicinal herbs for skincare by applying a data mining technique to investigate associations between medicinal herbs and skincare-related functions based on 26 skin-related keywords (SRKs) from Donguibogam texts as classical texts. The SRKs as medicinal properties were set up by several experts from the classical texts. Using a term frequency-inverse document frequency approach, they mined and extracted 52 candidate medicinal herbs by assessing herbal characteristics on the co-occurrence frequencies in which each candidate medicinal herb had least one of the 26 SRKs with tf-idf index *p*-values < 0.05 without concerning the high dimensional and correlated features of skin-related terms as medicinal herb properties. Their results showed that only 46 herbs out of 52 candidate medicinal herbs had skincare-related effects by employing bio-medicinal evaluation.

Yoo et al. (2020) proposed a deep learning-based approach to identify the medicinal uses (which were a part of medicinal properties) of natural compounds exploiting massive and heterogeneous drug and natural compound data involving structured and unstructured data from which they generated the three main feature groups: latent knowledge features (about 101 features) by text mining, molecular interaction features from protein-protein interactions (as a structured data base) via principal component analysis (PCA) to reduce the protein feature dimensionality from 4,487 to 285 features, and chemical property features (about 300 features) containing physiological and physicochemical properties with feature scaling by applying *Z*-score normalization. The result of an average AUROC value of their proposed method of identifying the medicinal uses in diseases related to the natural compounds was 0.90.

Zhang et al. (2020) assigned two different labels for the named-entity tagging scheme on the traditional Chinese medicine (TCM) book: a named-entity type label and a “Tie or Break” label, for neural network learning of the named-entity type prediction and the named-entity boundary detection, respectively. The results of named entity classification based on a multiclass model, i.e., Symptom, Chinese Medicine, Prescription, etc., had precision, recall and an F1 scores of 0.73, 0.67, and 0.70, respectively.

Jia et al. (2022) continued working on the named-entity recognition (NER) task by proposing a span-level distantly supervised approach to extract TCM medical entities from the TCM book by using a simple multilayer neural network as a classifier for multiclass classification, where the distant supervision used knowledge bases, domain ontologies and journals to automatically generate annotated datasets. Their proposed method was able to correctly classify the named entities with an F1 score of 0.77. Therefore, the previous works (Yoo et al., 2020; Jia et al., 2022) on determining named entities, e.g., Chinese medicines, applied the neural network learning technique by manual class labeling (Yoo et al., 2020) and automatic class labeling (Jia et al., 2022) to determine the named entities while the *mp*_*j*_ features were automatically determined by mainly using HerbMed and dictionaries.

Braik et al. (2023) worked on the feature selection by applying the basic capuchin search algorithm (CSA) for lowering the feature dimensionality in ML tasks for classification purposes on various data sets, including COVID-19 dataset as the structure data. They proposed using three methods of exponential CSA, power CSA, and S-shaped CSA, based on binary data to improve CSA in selecting the features with time consuming. One of their classification results on the COVID-19 datasets was an average accuracy rate of 95.9% after applying CSA with the low fitness value of 0.04. Their Feature selection techniques can reduce the dimensionality of the features but loss of information whereas the dimensionality reduction by SEM as wrapper-based method proposed in our research aim to minimize information loss.

Pechsiri and Piriyakul (2016) worked on automatically extracting a semantic relation between two event-explanation groups as a Problem-Solving relation, for example a DiseaseSymptom-Treatment relation from hospital-web-board documents. Each Problem or DiseaseSymptom group and each Solving or Treatment group were represented by a vector of problem-event features and a vector of solving-event features, respectively, where each event feature relied on a verb phrase expression. The feature extraction was based on the word cooccurrence extraction where the word cooccurrence consisted of two words (one word was a verb; and another word was a word right after the verb) to represent each event feature. They applied the simple k-means clustering method to object clustering and feature clustering for reducing the object and feature dimensions before learning the relation by Naïve Bayes. Their F1 score of the a DiseaseSymptom-Treatment relation extraction was 0.81.

The previous studies determined either the relation/association between the medicinal plants and the medicinal properties or the phytochemical activities related to the diseases with direct relation, indirect relation as a transitive relation, or part of a series effects without concerning the high dimensional and correlated features except (Yoo et al., 2020; Braik et al., 2023; Pechsiri and Piriyakul, 2016), and the grouped semantic-feature relation as the common relation. However, the pp-mpG relation extraction as the grouped semantic-feature relation extraction involves the high dimensional and correlated features from the documents to represent the medicinal-plant property knowledge on social media, which results in both potential remedy common ailments in the form of primary or supplementary treatments and using the medicinal plants as alternative medical therapy.

## 3 Methodology

**Figure 3:**
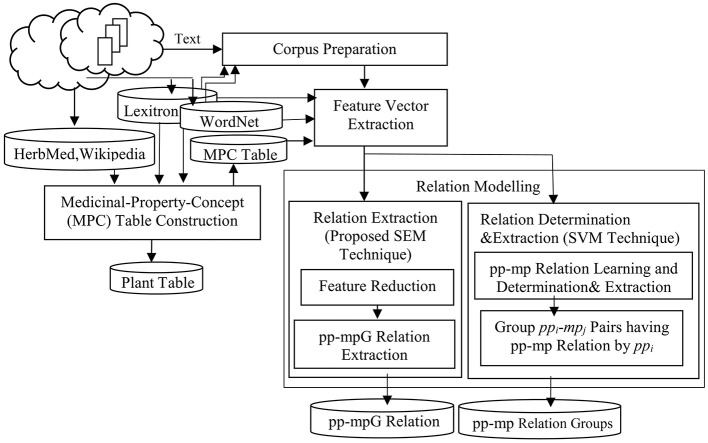
A system framework of extracting the pp-mpG relations by SEM and pp-mp relations by SVM.

There are four main steps within our framework: Corpus Preparation, Medicinal-Property-Concept (MPC) Table Construction, Feature Vector Extraction, and Relation Modeling. Relation modeling consists of two difference techniques, Extraction by Proposed SEM Technique (which consists of Feature Reduction, and pp-mpG Relation Extraction), and Relation Determination & Extraction by SVM technique (which consists of pp*-*mp Relation Learning and Determination & Extraction and Group *pp*_*i*_-*mp*_*j*_ Pairs having pp-mp Relations by *pp*_*i*_; see Figure 3).

### 3.1 Corpus preparation

From a total of 128 Thai research organization websites, this research selected 97 units using simple random sampling to ensure adequate statistical representation with an error rate not exceeding 5% (*n* ≥ N/(1+N^*^e^2^)), employing this approach due to the homogeneous nature of the sample units. The Thai-herbal-plant corpus preparation involved extracting 20,000 Elementary Discourse Units (EDUs) from 259 downloaded documents sourced from pharmacy academic websites (Table A.2), encompassing 50 distinct herbal-name concepts (*h*_*k*_) predominantly related to COVID-19 symptoms and Thai cooking ingredients, with document sizes varying from 20 to 130 EDUs per document. The corpus was strategically partitioned into two components: 5,000 EDUs from 64 documents for corpus behavior study and 15,000 EDUs from 195 documents for *mp*_*j*_ feature vector extraction, specifically targeting potential disease remedies analysis excluding body nourishment considerations, incorporating relation extraction using both the proposed SEM technique and the SVM technique. The preprocessing pipeline employed Thai word segmentation tools (Sudprasert and Kawtrakul, 2003) and named-entity recognition tools (Chanlekha and Kawtrakul, 2004; Tongtep and Theeramunkong, 2010) based on Lexitron and WordNet lexical resources, followed by EDU segmentation (Chareonsuk et al., 2005; Ketui et al., 2013) to achieve standardized discourse unit identification across the corpus for subsequent analytical procedures.

### 3.2 Medicinal-property-concept (MPC) table construction

It is necessary to collect the sets of terminal symbols, i.e., the Verb_weak_, Verb_strong_, Adv, Adj, and Noun sets as shown in Figure 2a, by the following method to construct the MPC table based on the WCPattern.

It is necessary to collect the sets of terminal symbols, i.e., the Verb_weak_, Verb_strong_, Adv, Adj, and Noun sets as shown in Figure 2a, by the following method to construct the MPC table based on the WCPattern.

a) The elements of the Verb_strong_, Verb_weak_, Noun, Adj, and Adv sets including the concepts of the elements in Figure 2a, are prepared and collected from the EDU_tmt_ expressions of HerbMed/Medical-Symptom Term List after word segmentation (see Table 1 presenting examples of herbal terms with medicinal-property concepts from HerbMed and Table 2 presenting terms with medical-symptom concepts from the Medical-Symptom Term List). The concepts of the segmented words as the element concepts after stemming words and eliminating stop words are determined by the Thai-to-English translation based on Lexitron and WordNet, as shown in Tables 1, 2. If any entities from Tables 1, 2 have the same element concept, they will be collected as one entity, e.g., “*aperient*” and “*laxative*” in Table 1. The examples in Tables 1, 2 also include the phonetic expression by http://translate.google.com/ (accessed on 23 August 2024).
b) If there is more than one sense/concept of an element from the Thai-to-English translation by Lexitron, we use WordNet to select the sense that contains the following elements of wrdSet, {“*patient*,” “*healthy*,” “*illness*,” “*body*,” “*wound*”}, on its description by WordNet. For example, “(ถ่าย/T̄h̀āy)/V_strong_” has three senses by Lexitron, “โยกย้าย/*transfer*,” “ถ่ายอุจจาระ/*excrete*,” and “ถ่ายภาพ/*photograph*.” The “*excrete*” sense is selected by the system because the description of “*excrete*” by WordNet is “*excrete, egest, eliminate, pass*—(*eliminate from the* *body*; ‘*Pass a kidney stone*'),” which contains “*body*” as the element of wrdSet.

**Table 1:** Example: Herbal terms with medicinal-property concepts from HerbMed are translated to Thai as EDU_tmt_ expressions by Lexitron.

**Herbal terms with medicinal-property concepts**	**EDU_tmt_**	**EDU_tmt_ including word segmentation**	**Segmented-word concept as element concept by Thai-to-English transalation based on Lexitron and WordNet**
*Antidiarrhetic*	แก้,รักษาอาการท้องเสีย/K$\hat{\text{æ}}$,Rạk$\bar{\text{s}}$′ā xākār tĥxng$\bar{\text{s}}$eī	((แก้,รักษา/K$\hat{\text{æ}}$,Rạk$\bar{\text{s}}$′ā)/V_strong_ ((อาการ/xākār)/Noun (ท้องเสีย/tĥxng$\bar{\text{s}}$eī)/V_strong_)/NP2)VP	(K$\hat{\text{æ}}$,Rạk$\bar{\text{s}}\prime$ā/*cure*)/V_strong_, (xākār/*symptom*)/Noun, (tĥxng$\bar{\text{s}}$eī/*have diarrhea*)/V_strong_
*Antiemetic*	ยาแก้อาเจียน/Yā k$\hat{\text{æ}}$ khlụ̄̀ns$\overset{⌢}{ị}$l$\hat{\text{æ}}$a xāceīyn	((ยา/Yā)/Noun)/NP1 ((แก้/k$\hat{\text{æ}}$)/(อาเจียน/xāceīyn)/V_strong_)/VP	(ยา/Yā)/Noun, (k$\hat{\text{æ}}$/*cure*)/V_strong_ (xāceīyn/*vomit*)/V_strong_
*Antihemorrhagic*	ยาห้ามเลือด/Yāh̄ ^āmle$\bar{\underset{˙}{\text{u}}}$xd; ยาแก้เลือดออก/Yā k$\hat{\text{æ}}$ le$\bar{\underset{˙}{\text{u}}}$xd xxk	((ยา/Yā)/Noun)/NP1 ((ห้าม/h̄ ^ām)/V_strong_ (เลือด/le$\bar{\underset{˙}{\text{u}}}$xd)/Noun)/VP; ((ยา/Yā)/Noun)/NP1 ((แก้/k$\hat{\text{æ}}$)/V_strong_ (เลือดออก/le$\bar{\underset{˙}{\text{u}}}$xd xxk)/V_strong_)/VP	(Yā/*medicine*)/Noun, (ām/*stop*)/V_strong_, (le$\bar{\underset{˙}{\text{u}}}$xd/*blood*)/Noun, (k$\hat{\text{æ}}$/*cure*)/V_strong_, (le$\bar{\underset{˙}{\text{u}}}$xd xxk/*bleed*)/V_strong_
*Anti-inflammatory*	ยาแก้อักเสบ/Yāk$\hat{\text{æ}}$xạk$\bar{\text{s}}$eb	((ยา/Yā)/Noun)/NP1 ((แก้/k$\hat{\text{æ}}$)/V_strong_ (อักเสบ/xạk$\bar{\text{s}}$eb)/V_strong_)/VP	(Yā/*medicine*)/Noun, (k$\hat{\text{æ}}$/*cure*)/V_strong_, (xạk$\bar{\text{s}}$eb/*inflame*)/V_strong_
*Antipruritic*	ยาลดอาการคัน/Yā Ld xākār khạn; *ยาแก้คัน*/Yā k$\hat{\text{æ}}$ khạn	((ยา/Yā)/Noun)/NP1 ((ลด/Ld)/V_strong_ (อาการ/xākār)/Noun (คัน/khạn)/V_strong_)/VP; ((ยา/Yā)/Noun)/NP1 ((แก้/k$\hat{\text{æ}}$)/V_strong_ (*คัน*/khạn)/V_strong_)/VP	(Yā/*medicine*)/Noun, (xākār/*symptom*)/Noun, (khạn/*itch*)/V_strong_, (k$\hat{\text{æ}}$/*cure*)/Vstrong
*Antipyretic*	ยาลดไข้/Yā ld $\bar{\text{k}}$h$\overset{⌢}{ị}$	((ยา/Yā)/Noun)/NP1 ((ลด/Ld)/V_strong_ (ไข้/$\bar{\text{k}}$h$\overset{⌢}{ị}$)/Noun)/VP	(Yā/*medicine*)/Noun, (Ld/*reduce*)/V_strong_, ($\bar{\text{k}}$h$\overset{⌢}{ị}$/*fever*)/Noun
*Antitussive*	ยาบรรเทาอาการไอ/Yā brrtheā; xākār xị; ยาแก้ไอ/Yā k$\hat{\text{æ}}$ xị	((ยา/Yā)/Noun)/NP1 ((บรรเทา/brrtheā)/V_strong_ (อาการ/xākār)/Noun (ไอ/xị)/V_strong_)/VP; ((ยา/Yā)/Noun)/NP1 ((แก้/k$\hat{\text{æ}}$)/V_strong_ (ไอ/xị)/V_strong_)/VP	(Yā/*medicine*)/Noun, (brrtheā/*relieve*)/V_strong_, (xākār/*symptom*)/Noun, (xị/*cough*)/V_strong_, (/k$\hat{\text{æ}}$/*cure*)/V_strong_
*Antiviral*	ยาต้านเชื้อไวรัส/Yā $\hat{\text{t}}$ān che$\hat{\bar{\underset{˙}{\text{u}}}}$x wịrạ$\bar{\text{s}}$; ยาต้านไวรัส/Yā $\hat{\text{t}}$ān wịrạ$\bar{\text{s}}$	((ยา/Yā)/Noun)/NP1 ((ต้าน/$\hat{\text{t}}$ān)/V_strong_ (เชื้อไวรัส/che$\hat{\bar{\underset{˙}{\text{u}}}}$x wịrạ$\bar{\text{s}}$)/Noun)/VP; ((ยา/Yā)/Noun)/NP1 ((ต้าน/$\hat{\text{t}}$ān)/V_strong_ (ไวรัส/wịrạ$\bar{\text{s}}$)/Noun)/VP	(Yā/*medicine*)/Noun, ($\hat{\text{t}}$ān/*resist*)/V_strong_, (che$\hat{\bar{\underset{˙}{\text{u}}}}$x wịrạ$\bar{\text{s}}$/*virus*)/Noun, (wịrạ$\bar{\text{s}}$/*virus*)/Noun
*Aperient*/*laxative*	เป็นยาระบาย/Yā rabāy; ยาถ่าย/Yā t̄h̀āy; ยาแก้ท้องผูก/Yā k$\hat{\text{æ}}$ tĥxng$\bar{\text{p}}$hūk	((เป็น/Pĕn)/V_weak_ (ยา/Yā)/Noun (ระบาย/rabāy)/V_strong_)/VP; ((ยา/Yā)/Noun)/NP1 ((ถ่าย/t̄h̀āy)/V_strong_)/VP; ((ยา/Yā)/Noun)/NP1 ((แก้/k$\hat{\text{æ}}$)/V_strong_ (ท้องผูก/tĥxng$\bar{\text{p}}$hūk)/V_strong_)/VP	((Pĕn/be)/V_weak_ (Yā/*medicine*)/Noun), (Yā/*medicine*)/Noun, (rabāy/*release*)/V_strong_, (k$\hat{\text{æ}}$/*cure*)/V_strong_, (t̄h̀āy/*excrete*)/V_strong_), (tĥxng$\bar{\text{p}}$hūk/*be constipated*)/V_strong_
*Astringent*	เป็นยาฝาดสมาน/Pĕn yā f̄ād $\bar{\text{s}}$mān; ยาสมานแผล/Yā $\bar{\text{s}}$mān $\bar{\text{p}}$h$\hat{\text{æ}}$l	((เป็น/Pĕn)/V_weak_ (ยา/yā)/Noun (ฝาดสมาน/f̄ād $\bar{\text{s}}$mān)/Adj)/NP1)/VP; ((ยา/Yā)/Noun)/NP1 ((สมาน/$\bar{\text{s}}$mān)/V_strong_ (แผล/$\bar{\text{p}}$h$\hat{\text{æ}}$l)/Noun)/VP	((Pĕn/be)/V_weak_ (Yā/*medicine*)/Noun), (Yā/*medicine*)/Noun, (f̄ād $\bar{\text{s}}$mān/*astrictive*)/Adj, ($\bar{\text{s}}$mān/*heal up*)/V_strong_, ($\bar{\text{p}}$h$\hat{\text{æ}}$l/*wound*)/Noun
*Carminative*	ยาขับลม/Yā $\bar{\text{k}}$hạb lm	((ยา/Yā)/Noun)/NP1 ((ขับ/$\bar{\text{k}}$hạb)/V_strong_ (ลม/lm)/Noun)/VP	(Yā/*medicine*)/Noun, ($\bar{\text{k}}$hạb/*expel*)/V_strong_, (lm/*air,gas*)/Noun
*Diaphoretic*/*hidrotic*/*sudorific*	สารขับเหงื่อ/S̄ār $\bar{\text{k}}$hạb $\bar{\text{h}}$engụ̄̀x; ขับเหงื่อ/$\bar{\text{k}}$hạb $\bar{\text{h}}$engụ̄̀x	((สาร/S̄ār)/Noun)/NP1 ((ขับ/$\bar{\text{k}}$hạb)/V_strong_ (เหงื่อ/$\bar{\text{h}}$engụ̄̀x)/Noun)/VP; ((ขับ/$\bar{\text{k}}$hạb)/V_strong_ (เหงื่อ/$\bar{\text{h}}$engụ̄̀x)/Noun)/VP	(S̄ār/*substance*)/Noun, ($\bar{\text{k}}$hạb/*expel*)/V_strong_, ($\bar{\text{h}}$engụ̄̀x/*sweat*)/Noun
*Hypoglycemant*	ยาลดน้ำตาลในเลือด/Yā ld $\hat{\text{n}}$ảtāl nı le$\bar{\underset{˙}{\text{u}}}$xd	((ยา/Yā)/Noun)/NP1 ((ลด/Ld)/V_strong_ (น้ำตาล/$\hat{\text{n}}$ảtāl)/Noun (ใน/nı)/Prep (เลือด le$\bar{\underset{˙}{\text{u}}}$xd)/Noun)/VP	(Yā/*medicine*)/Noun, (Ld/*reduce*)/V_strong_, ($\hat{\text{n}}$ảtāl/*sugar*)/Noun, (le$\bar{\underset{˙}{\text{u}}}$xd/*blood*)/Noun
*Vulnerary*	รักษาบาดแผล/rạk$\bar{\text{s}}$′ā bād$\bar{\text{p}}$h$\hat{\text{æ}}$l; สิ่งรักษาบาดแผล/S̄ìng rạk$\bar{\text{s}}$′ā bād$\bar{\text{p}}$h$\hat{\text{æ}}$l	((รักษา/Rạk$\bar{\text{s}}$′ā)/V_strong_ (บาดแผล/bād$\bar{\text{p}}$h$\hat{\text{æ}}$l)/Noun)VP; ((สิ่ง/S̄ìng)/Noun)/NP1 ((รักษา/Rạk$\bar{\text{s}}$′ā)/V_strong_ (บาดแผล/bād$\bar{\text{p}}$h$\hat{\text{æ}}$l)/Noun)VP	(Rạk$\bar{\text{s}}$′ā/*cure*)/V_strong_, (bād$\bar{\text{p}}$h$\hat{\text{æ}}$l/*wound*)/Noun, (S̄ìng/*thing*)/Noun
**….**.	**….**.	**….**.	**….**.

**Table 2:** Example: terms with medical-symptom concepts from medical-symptom term list (Wikipedia) are translated to Thai as EDU_tmt_ expressions by Lexitron.

**Terms with medical-symptom concepts**	**EDU_tmt_**	**EDU_tmt_ including word segmentation**	**Segmented-word concept as element concept by Thai-to-English transalation based on Lexitron and WordNet**
*Epistaxis*/*nosebleed*	เลือดกำเดาไหล/Le$\bar{\underset{˙}{\text{u}}}$xd kảdeā $\bar{\text{h}}$ịl; กำเดาไหล/ kảdeā $\bar{\text{h}}$ịl	((เลือดกำเดา/Le$\bar{\underset{˙}{\text{u}}}$xd kảdeā)/Noun)/NP ((ไหล/$\bar{\text{h}}$ịl)/V_strong_)/VP; ((กำเดา/ kảdeā)/Noun)/NP ((ไหล/$\bar{\text{h}}$ịl)/V_strong_)/VP	(Le$\bar{\underset{˙}{\text{u}}}$xd kảdeā/*nose blood*)/Noun, (kảdeā/*nose blood*)/Noun, ($\bar{\text{h}}$ịl/*come out*)/V_strong_
*Faint*	วิงเวียน/Wingweīyn; เป็นลม/pĕn lm	(วิงเวียน/Wingweīyn)/V_strong_; (เป็นลม/pĕn lm)/V_strong_	(Wingweīyn/*faint*,*dizzy*)/V_strong_, (pĕn lm/*faint*,*dizzy*)/V_strong_
*Flatulence*	อาการท้องเฟ้อ/Xākār tĥxngfêx	((อาการ/Xākār)/Noun (ท้องเฟ้อ/tĥxngfêx)/Adj)/NP	(Xākār/*symptom*)/Noun, (tĥxngfêx/*flatulence*)/Adj
*Malaise*	อ่อนเพลีย/$\overset{`}{\text{X}}$xnphelīy	(อ่อนเพลีย/$\overset{`}{\text{X}}$xnphelīy)/V_strong_	($\overset{`}{\text{X}}$xnphelīy/*be tired*)/V_strong_
*Sore throat*	เจ็บคอ/Cĕb khx	((เจ็บ/Cĕb)/V_strong_ (คอ/khx)/Noun)/VP	(Cĕb/*be sore*)/V_strong_, (khx/*throat*)/Noun
……………	……………	……………	……………

As shown in Table 1, we determine the word-co expressions based on WCPattern on EDU_tmt_, after the word segmentation followed by stemming words and eliminating stop words. The determined word-co expressions on EDU_tmt_ and their corresponding term in the Herbal Terms with Medicinal-Property Concepts column are the wrd-CoExp elements and the *mpc* elements, respectively (wrd-CoExp∈word-CoExpressions, *mpc*∈ MedicinalPropertyConcepts) as shown in Table 3 with the phonetic expression.

**Table 3:** Show word-co expressions on the segmented EDU_tmt_ expressions aligned with medicinal property concepts from HerbMed.

**MedicinalPropertyConcepts (*mpc* ∈MedicinalPropertyConcepts)**	**word-CoExpressions (wrd-CoExp ∈word-CoExpressions)**
**….**.	**….**.
*Antidiarrhetic*	(แก้,รักษา/k$\hat{\text{æ}}$,Rạk$\bar{\text{s}}$′ā/*cure*)+ (ท้องเสีย/tĥxng$\bar{\text{s}}$eī/*have diarrhea*) ➔“รักษา+ท้องเสีย”; “แก้+ท้องเสีย”
*Antiemetic*	(แก้/k$\hat{\text{æ}}$/*cure*)+ (อาเจียน/xāceīyn/*vomit*) ➔“แก้+อาเจียน”
*Antihemorrhagic*	(ห้าม/h̄ām/*stop*)+ (เลือด/le$\bar{\underset{˙}{\text{u}}}$xd/*blood*); (แก้/k$\hat{\text{æ}}$/*cure*)+ (เลือดออก/le$\bar{\underset{˙}{\text{u}}}$xd xxk/*bleed*) ➔“ห้าม+เลือด,” “แก้+เลือดออก”
*Anti-inflammatory*	((แก้/k$\hat{\text{æ}}$/*cure*)+ (อักเสบ/xạk$\bar{\text{s}}$eb/*inflame*) ➔“แก้+อักเสบ”
*Antipruritic*	(ลด/Ld/*reduce*)+ (คัน/khạn/*itch*); (แก้/k$\hat{\text{æ}}$/*cure*)+ (คัน/khạn/*itch*) ➔“ลด+คัน”; “แก้+คัน”
**….**.	**….**.

In addition to Table 2, each word sequence (*w*_1_, *w*_2_, *w*_3_) of WCPattern that is determined from EDU_tmt_ after the word segmentation followed by stemming words and eliminating stop words is then added *ss* (where *ss* ∈ SVCsub; SVCsub ⊂ SVC; SVCsub = {“แก้,รักษา/*cure, treat, remedy*,” “ห้าม/*stop*,” “เพิ่ม/*increase*,” “ลด/*reduce*,” “บรรเทา/*relieve*,” “ขจัด/*expel, anti-*,” “ทำให้/*cause*”}) in front of both the word sequence (*ss* + “*w*_1_, *w*_2_, *w*_3_”) and its corresponding term in the “Terms with Medical-Symptom Concepts” column of Table 2 to form a wrd-CoExp element and a *mpc* element, respectively, as shown in Table 4.

**Table 4:** Show *ss* plus word-co expressions on the segmented EDU_tmt_ expressions aligned with *ss* plus medicinal property concepts from medical-symptom concept term in Wikipedia where *ss* ∈ SVCsub; SVCsub ⊂ SVC.

**Medicinal property concepts (*mpc*) from medical symptom concept**	**word-CoExpressions (wrd-CoExp ∈word-CoExpressions)**
**….**.	**….**.
*ss*+*flatulence*	*ss*+(ท้องเฟ้อ/tĥxngfêx)➔“*ss*+ท้องเฟ้อ”
*ss*+*faint*	*ss*+(วิงเวียน/Wingweīyn); *ss*+(เป็นลม/pĕn lm) ➔“*ss*+วิงเวียน”; “*ss*+เป็นลม”
*ss*+*epistaxis*/*nosebleed*	*ss*+(เลือดกำเดา/Le$\bar{\underset{˙}{\text{u}}}$xd kảdeā) + (ไหล/$\bar{\text{h}}$ịl); *ss*+(กำเดา/kảdeā) + (ไหล/$\bar{\text{h}}$ịl) ➔“*ss*+เลือด+กำเดาไหล”; “*ss*+เกำเดา+ไหล”; “*ss*+เลือดกำเดา”; “*ss*+เกำเดา”
*ss*+*nausea*	*ss*+(คลื่นไส้/khlụ̄̀ns$\overset{⌢}{ị}$/*nauseate*) ➔ “แก้+คลื่นไส้”
*ss*+*sore throat*	*ss*+((เจ็บ/Cĕb) + (คอ/khx) ➔“*ss*+เจ็บ+คอ”
**….**.	**….**.

Thus, Table 3 is appended by the formations of the *mpc* elements and the corresponding wrd-CoExp elements from Table 4 to generate the MPC table (Table 5) including the phonetic expressions. Further, if any entities from Table 5 have the same wrd-CoExp element, they will be collected into one entity for redundancy elimination.

**Table 5:** The medicinal-property-concept (MPC) table.

**MedicinalPropertyConcepts (*mpc* ∈MedicinalPropertyConcepts)**	**word-CoExpressions (wrd-CoExp ∈word-CoExpressions)**
**….**.	**….**.
*Antidiarrhetic*	(แก้,/k$\hat{\text{æ}}$/*cure*) + (ท้องเสีย/tĥxng$\bar{\text{s}}$eī/*have diarrhea*) = “แก้+ท้องเสีย”; (รักษา/k$\hat{\text{æ}}$,Rạk$\bar{\text{s}}$′ā/*cure*) + (ท้องเสีย/tĥxng$\bar{\text{s}}$eī/*have diarrhea*) = “รักษา+ท้องเสีย”
*Antiemetic*	(แก้/k$\hat{\text{æ}}$/*cure*) + (อาเจียน/xāceīyn/*vomit*) = “แก้+อาเจียน”
*Antihemorrhagic*	(ห้าม/h̄ām/*stop*) + (เลือด/le$\bar{\underset{˙}{\text{u}}}$xd/*blood*) = “ห้าม+เลือด”; (แก้/k$\hat{\text{æ}}$/*cure*) + (เลือดออก/le$\bar{\underset{˙}{\text{u}}}$xd xxk/*bleed*) = “แก้+เลือดออก”
*Anti-inflammatory*	((แก้/k$\hat{\text{æ}}$/*cure*) + (อักเสบ/xạk$\bar{\text{s}}$eb/*inflame*) = “แก้+อักเสบ”
*Antipruritic*	(ลด/Ld/*reduce*) + (คัน/khạn/*itch*) = “ลด+คัน”; (แก้/k$\hat{\text{æ}}$/*cure*) + (คัน/khạn/*itch*) = “แก้+คัน”
*Antipyretic*	((ลด/Ld/*reduce*) + (ไข้/$\bar{\text{k}}$h$\overset{⌢}{ị}$/*fever*) = “ลด+ไข้”
*Antitussive*	(บรรเทา/brrtheā/*relieve*) + (ไอ/xị/*cough*) = “บรรเทา+ไอ”; (แก้/k$\hat{\text{æ}}$/*cure*) + (ไอ/xị/*cough*) = “แก้+ไอ”
*Antiviral*	(ต้าน/$\hat{\text{t}}$ān/*resist*) + (เชื้อไวรัส/che$\hat{\bar{\underset{˙}{\text{u}}}}$x wịrạ$\bar{\text{s}}$/*virus*)= “ต้าน+เชื้อไวรัส”; (ต้าน/$\hat{\text{t}}$ān/*resist*) + (ไวรัส/wịrạ$\bar{\text{s}}$/*virus*) = “ต้าน+ไวรัส”
*Aperient, laxative*	((เป็น/Pĕn) + (ยา/Yā)) + (ระบาย/rabāy/*release*) = “เป็น+ยา+ระบาย”; ((เป็น/Pĕn) + (ยา/Yā)) + (ถ่าย/t̄h̀āy/*excrete*) = “เป็น+ยา+ ถ่าย”; (แก้/k$\hat{\text{æ}}$/*cure*) + (ท้องผูก/tĥxng$\bar{\text{p}}$hūk/*be constipated*) = “แก้+ท้องผูก”
*Astringent*	((เป็น/Pĕn/*be*) + (ยา/Yā/*medicine*)) + (ฝาดสมาน/f̄ād $\bar{\text{s}}$mān/*astrictive*) = “เป็น+ยา+ฝาดสมาน”; (สมาน/$\bar{\text{s}}$mān/*heal up*) + (แผล/$\bar{\text{p}}$h$\hat{\text{æ}}$l/*wound*) = “สมาน+แผล”
*Carminative*	(ขับ/$\bar{\text{k}}$hạb/*expel*) + (ลม/lm/*air,gas*) = “ขับ+ลม”; (ผายลม/$\bar{\text{P}}$hāylm/*pass gas, fart*) = “ผายลม”
*Diaphoretic*/*hidrotic*/*sudorific*	(ขับ/$\bar{\text{k}}$hạb/*expel*) + (เหงื่อ/$\bar{\text{h}}$engụ̄̀x/*sweat*) = “ขับ+เหงื่อ”
*Hypoglycemant*	(ลด/Ld/*reduce*) + (น้ำตาล$\hat{\text{n}}$ảtāl/*sugar*) + (เลือด/le$\bar{\underset{˙}{\text{u}}}$xd/*blood*) = ลด+น้ำตาล+เลือด”
*Vermifuge*	(ขับ/$\bar{\text{k}}$hạb/*expel*) + (พยาธิ/phyāṭhi/*parasite*) = “ขับ+พยาธิ”; (ฆ่า/ḳh̀ā/*kill*) + (พยาธิ/phyāṭhi/*parasite*) = “ฆ่า+พยาธิ”; (ถ่าย/t̄h̀āy/*excrete*) + (พยาธิ/phyāṭhi/*parasite*) = “ถ่าย+พยาธิ”
*Vulnerary*	(รักษา/Rạk$\bar{\text{s}}$′ā/*cure*) + (บาดแผล/bādphæl/*wound*) = “รักษา+บาดแผล”; (รักษา/Rạk$\bar{\text{s}}$′ā/*cure*) + (แผล/$\bar{\text{p}}$hæl/*wound*) = “รักษา+แผล”
**….**.	**….**.
*ss*+*flatulence*	*ss*+(ท้องเฟ้อ/tĥxngfêx) = “*ss*+ท้องเฟ้อ”
*ss*+*faint*	*ss*+(เป็นลม/pĕn lm) = “*ss*+เป็นลม”; *ss*+(วิงเวียน/Wingweīyn) = “*ss*+วิงเวียน”
*ss*+*epistaxis*/*nosebleed*	*ss*+(เลือดกำเดา/Le$\bar{\underset{˙}{\text{u}}}$xd kảdeā) + (ไหล/$\bar{\text{h}}$ịl) = “*ss*+เลือดกำเดา+ไหล”; *ss*+(กำเดา/kảdeā) + (ไหล/$\bar{\text{h}}$ịl) = “*ss*+เกำเดา+ไหล”; *ss*+(กำเดา/kảdeā) = “*ss*+เกำเดา”
*ss*+*nausea*	*ss*+(คลื่นไส้/khlụ̄̀ns$\overset{⌢}{ị}$/*nauseate*) = “*ss*+คลื่นไส้”
*ss*+*sore throat*	*ss*+((เจ็บ/Cĕb) + (คอ/khx) = “*ss*+เจ็บ+คอ”
**….**.	**….**.

### 3.3 Feature vector extraction

The example of the corpus preparation from Section 3.1 for Figure 2a is shown in the following for the feature vector extraction.

Example of Corpus Preparation (where the [..] sympbol means ellipsis; the underline on an EDU expression shows a word-co expression based on WCPattern of the corpus's EDU expression).

File name: “กะเพรา/*Thai basil*”

Sub-topic name: (ใบ/Leaf)/NP1

EDU1: “([((ใบ/*leaf*)/Noun)/NP1] ((มี/*have*)/Verb_weak_ (รส/ *taste*/Noun) (เผ็ดร้อน/*spicy*)/Adj)/VP)/EDU”
“[*A leaf*] *has a spicy taste*.”
EDU2: “(((ต้ม/*boil*)/Verb_strong_ (เอา/*get*)/Verb_werk_) (น้ำดื่ม/ *drinking water*)/Noun)/VP)/EDU”
“*Boil to get the water for drinking*.”
EDU3: “(([(ดื่ม/drink)/V_erbstrong_] (เป็น/be)/Verb_weak_ (ยา/ medicine)/Noun (ขับ/*pass*)/Verb_strong_(ลม/gas)/Noun)/VP)/EDU”
“[*Drink*] *as a carminative*.”
EDU4: “(((แก้/treat)/Verb_strong_ (ท้องอืด, ท้องเฟ้อ/bloat)/Adv)/VP)/EDU”
“*Treat Bloating or flatulence*.”
EDU 5: “(((แก้/treat)/Verb_strong_ (ปวด/pain)/Verb_strong_ (ท้อง/abdomend)/Noun)/VP)/EDU”
“*Relieve stomach pain*.”
EDU 6: “(((แก้/treat)/Verb_strong_ (จุกเสียด/be colic)/Verb_strong_)/VP)/EDU”
“*Cure colic*.”
EDU7: “(((แก้/treat)/Verb_strong_ (คลื่นเหียนอาเจียน, คลื่นไส้/ *nauseate**)/Verb_strong_)/* VP)/EDU”
“*Cure nausea and vomit*.”
EDU 8: “(((ขับ/excrete)/Verb_strong_ (เสมหะ/*phlegm**)Noun)/VP*)/EDU”
“*Excrete phlegm*.”
  …………………….
EDU12: “(((นำ/*use*)/Verb_weak_ (ใบ/leaf)/Noun (สด/*fresh*)/Adj (บีบคั้น/*squeeze*)/Verb_strong_ (น้ำ/*water*)/Noun)/VP)/EDU”
“*Use/bring fresh leaves to squeeze juice*.”
EDU13: “(((ทา/apply)/Verb_strong_)/VP)/EDU”
“*Apply*.”
EDU14: “(((แก้/cure)/Verb_strong_ (โรคผิวหนัง/*Skin Disease**)/Noun)/* VP)/EDU”
“*Cure skin disease*.”

**Figure 4:**
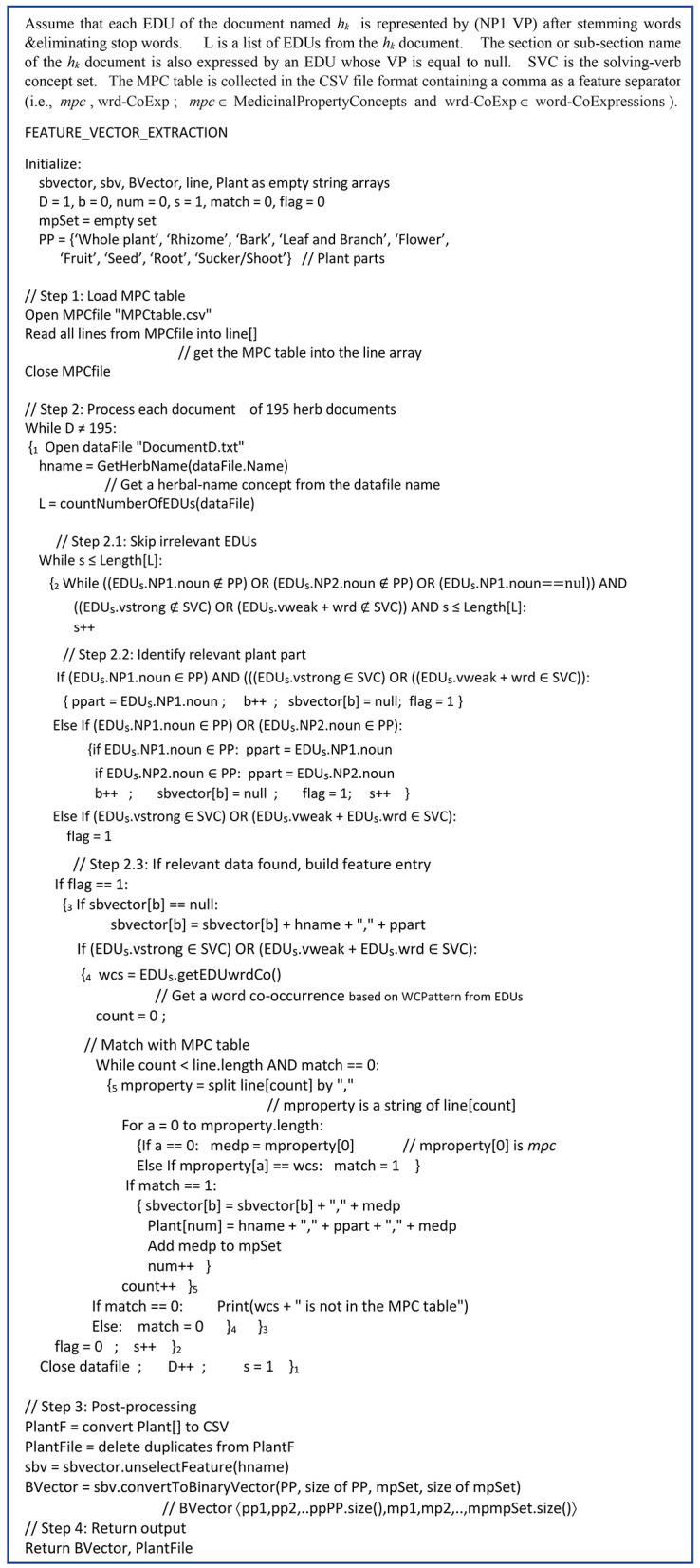
The FeatureVector_Extraction algorithm.

Regarding Figure 4, the FeatureVector_Extraction algorithm consists of the following 4 main steps after initializing the symbolic-feature vectors (such as sbvector, sbv, line, Plant, and Bvector) represented by an array of a string vector, e.g., sbvector_[b]_; *b* = 1,2,..,*numofsbvectors*) that consists of the determined features as follows: the herbal-name concept (*hname*) feature the plant-part concept (*ppart*) feature including the medicinal-property concept (*medp*) features of each *ppart* feature from the *hname* document:

Step1 Load MPC table: this step is to load the MPC table file into line_[]_.

Step2 Process each document of 195 herb document:

While numberOfDocuments (D) is not equal to 195

// this step is carried out to extract several symbolic-feature vectors (sbvector) from the 195 downloaded documents as the document files shown in the following steps within the while loop

Begin-WhileLoop-jnStep2

Open a data file of a herb document as DocumentD.txt

e.g., Basil1.txt, Basil2.txt, Ginger1.txt, etc. or the document topic names determined by named-entity recognition (Chanlekha and Kawtrakul, 2004; Tongtep and Theeramunkong, 2010) based on Lexitron.

After the *hname* feature is determined and extracted from the document file name in the corpus, L (the number of EDUs in the document file name) is determined.

Step2.1 Skip irrelevant EDUs within the current document:

While sLength[L]

Begin-WhileLoop-inStep2.1

Skip irrelevant EDUs of the current document

Step2.2 Identify relevant plant part:

- The *ppart* feature is determined from EDU_*s*_.NP1.noun/ EDU_*s*_NP2.noun (which is the noun (or Noun in Figure 2a) of NP1/NP2, respectively, in EDU_*s*_; *s* = 1, 2, ..., *numofEDUinAh*_*k*_*Document*) as the element of PP where
  PP = {“ทั้งต้น/*WholePlant*,” “ลำต้นใต้ดิน/*Rhizome*,” “เปลือกต้น/*Bark*,” “ใบและก้าน/*LeafandBranch*,” “ดอก/*Flower*,” “ผล/*Fruit*,” “เมล็ด/*Seed*,” “ราก/*Root*,” “หน่อ/*Shoot*”}.

- In addition, the section name or the sub-section name of the corpus document is also represented by an EDU_*s*_ where EDU_*s*_.VP (VP of EDU_*s*_) is equal to null.
- The determined *ppart* feature involves the various *medp* features on several EDU occurrences where one *medp* feature occurs in one EDU occurrence.
- SVC is used to identify a medicinal-property concept EDU_s_ to extract a *medp* feature in Step2.3. If the medicinal-property concept EDU_s_ is found, the flag is set to 1 and a *b* increment occurs.
  SVC = {“แก้,รักษา/*cure*,*treat*,*remedy*,” “ห้าม/*stop*,” “เพิ่ม/*increase*,” “ลด/*reduce*,” “บรรเทา/*relieve*,” “ขับ/*release*,” “สมาน/*heel*,” “ขจัด/*expel, anti-*,” “ขับถ่าย,ถ่าย,ระบาย/*excrete*,” “เป็น/*be*+ยา/*medicine*,” “ช่วย/*help*+ขับ/*excrete*” “ใช้/*use-for*+รักษา/*remedy*”}

Step2.3 If relevant data found, build feature entry:

- This step of the *medp* feature extraction occurs if flag = 1.
- The *medp* feature is determined by extracting *wc*_*s*_ (which is EDU_*s*_.wc or a word-co expression based on WCPattern in EDU_*s*_). If MPC.wrd-CoExp has a string matching to *wc*_*s*_, then *medp* of *wc*_*s*_is the *mpc* of MPC.wrd-CoExp in line_[*count*]_..
- Add the feature entry into sbvector_[*b*]_
- Flag is set to 0; a *s* increment occurs.
- End-WhileLoop-inStep2.1

Close a data file; a D increment occurs; *s* is set to 1

End-WhileLoop-inStep2.

In conclusion, *sbvector*_[b]_, which is a symbolic vector, consists of the extracted features with the comma separator as follows: the *hname*/*h*_*k*_ feature and the *ppart*/*pp*_*i*_ feature including the *medp*/*mp*_*j*_ features of the corresponding *ppart*/*pp*_*i*_ feature. Moreover, the extracted features are also collected into Plant_[num]_ which consists of three attributes: the *h*_*k*_ feature, the *pp*_*i*_ feature, and the *mp*_*j*_ feature. Plant_[num]_ is used to provide the medicinal-plant name of the certain the pp-mpG relation.

Step3 Post processing

After all symbolic-feature vectors (sbvector) have been extracted from the corpus, all features of sbvector except the *h*_*k*_feature are converted into binary-feature vectors (BVector). BVector, represented by an array of binary vectors as BVector_[b]_ having *b* = 1,2,..,*numofBVectors*, consists of the following features: *pp*_*i*_ (*i* = 1,2,…, *numOfPlantPartConcepts* or 9) and *mp*_*j*_ [*j* = 1,2,…, *MedicinalPropertyConcepts* or *m* which is mpSet.size() and is equal to 88 (see Table A.3); and mpSet is a medicinal-property concept set].

Moreover, Plant_[num]_ is converted to a comma delimited file (a CSV file named “PlantF”). The redundancy occurrences in PlantF are eliminated to become the plant file named PlantFile.

Step4 Return output:

Return Bvector, PlantFile

### 3.4 Relation modeling

There are two relation modeling techniques for the relation extraction: SEM and SVM.

#### 3.4.1 Relation extraction (proposed SEM technique)

SEM combines aspects of factor analysis to reduce features in the first step and multiple regression into one comprehensive model in the second step as follow.

##### 3.4.1.1 Feature reduction

The extracted BVector_[b]_, < *pp*_1_,*pp*_2_,..*pp*_9_, *mp*_1_,*mp*_2_,..*mp*_*m*_>_[b]_, from the previous step contains a medicinal-property concept vector (MPvector_[b]_), < *mp*_1_,*mp*_2_,..*mp*_*m*_>_[b]_, with *m* = 88 as the high-dimensional feature space. The hierarchical factor analysis technique (Kim and Mueller, 1978; Brunner et al., 2011), a common factor analysis method at the first-order factor model, is then applied to reduce the *mp*_*j*_ features on MPvector by using covariance matrices to determine which variables have the highest correlation and then groups those variables together into a factor as follows. According to the extraction of the MPvector_[b]_ occurrences (with *b* = 1,2,..,617), the medicinal-property concept feature matrix (MPmatrix) is then rotated to group the medicinal-property concept features of the vector into separated feature groups with the minimum number of separated feature groups where each separated feature group is called “Fgroup_*z*_”; *z* = 1,2,...,*numofFeatureGroups* which is less than *m*. After the medicinal-property concept feature vector rotation, a feature loading weight from an eigenvector for the *mp*_*j*_ feature is determined according to Fgroup_*z*_. The high feature loading weight of *mp*_*j*_ to Fgroup_*z*_ infers that the correlation between *mp*_*j*_ and Fgroup_*z*_ is high. The different *mp*_*j*_ feature elements with the high feature loading weights in a certain Fgroup_*z*_ are wrapped to become a factor (called “Factor_*z*_”) including its factor score (called “FactorScore_*z*_”) determined by Equation 1 from the feature loading weights of the wrapped *mp*_*j*_ feature elements.


(1) $$\begin{array}{l} FactorScore_{z}=\;\overset{k_{z}}{\underset{j=1}{\sum }}w_{j}\left(\frac{x_{j}-{\bar{x}}_{j}}{SD_{j}}\right)\; \end{array}$$

where:

*w*_*j*_ is a feature loading weight of *mp*_*j*_ from an eigenvector in Factor_*z*_;

*mp*_*j*_ is an element of the medicinal-property concept feature within Factor_*z*_; *j* = 1,2,…,*k*_*z*_;

*k*_*z*_ is the number of different medicinal-property concept features in Factor_*z*_;

*x*_*c*_ is an original value of the number of each *mp*_*j*_ with its mean, ${\bar{x}}_{j}$, and standard deviation, SD_*j*_

At the first level of the factor analysis or the first-order factor model, the common factor analysis method (based on IBM SPSS Statistics for Windows, Version 21.0) is used to reduce the number of *m* features of the vector of the medicinal-property concept features by wrapping each *mp*_*j*_ feature element with the feature loading weight from the eigenvector ≥|0.4| based on our corpus within the corresponding Fgroup_*z*_ to become Factor_*z*_ (*z* = 1,2,..., *numofFeatureGroups* which is 15). Each Factor_*z*_ contains several elements of the different *mp*_*j*_ features. One *mp*_*j*_ feature can exist in only one Factor_*z*_. At the second level of the factor analysis or the higher-order factor model, Factor_1_, Factor_2_, …, Factor_15_ are grouped or wrapped by the common factor analysis method into core factors for the pp-mpG relation determination and extraction in the next step (pp-mpG relation extraction).

The application of Factor Analysis principles for dimensionality reduction of medicinal properties effectively addresses both high-dimensionality and redundancy issues, which constitute NP-hard computational problems; Alweshah et al., 2016, particularly within NLP contexts, while utilizing unsupervised data and offering superior advantages when integrated with Machine Learning approaches to examine plant component-medicinal property relationships through Structural Equation Modeling (SEM). This methodology delivers three critical computational cost advantages: first, enhanced computational time efficiency through initial Distributed Processing followed by Centralized Processing for result compilation and synthesis; second, significant relationship testing time reduction achieved by transforming the original number of medicinal property variables (*n*) to reduced factor dimensions (*k*, where *k*<*n*), exemplified by a seven-fold analysis time reduction when *n* = 21 and *k* = 3; and third, substantial reduction in supervised data processing time requirements and associated computational overhead. These combined computational efficiencies render this approach highly advantageous for large-scale medicinal property analysis applications while preserving analytical rigor, statistical validity, and predictive accuracy across diverse plant-based pharmaceutical research domains.

##### 3.4.1.2 pp-mpG relation extraction

Concerning (Schumacker and Lomax, 2004), structural equation modeling (SEM) serves as a robust statistical framework that encompasses multiple regression analysis and factor analysis, designed to unveil and elucidate relationships among antecedent and consequent factors (variables). SEM is used to analyze structural relationships, i.e., the pp-mpG relation. SEM analyzes the structural relationship between measured variables, i.e., the *pp*_*i*_ features, and latent constructs, i.e., the core factors. Additionally, SEM comprises two primary categories: variance-based SEM and covariance-based SEM (CB SEM). According to Petrovska (2012) and Parihar and Balekar (2016), explanations of plant parts related to medicinal properties, CB SEM is applied to provide direct relevance when examining the pp-mpG relation as the actual relation occurrences between the plant-part concept feature and the medicinal-property concept features.

The data analysis in this study falls into two distinct phases. In Step 1, we initiate a hierarchical feature reduction process, initially reducing 88 features to 15 factors. Subsequently, these 15 factors (denoted as AC1 to AC15) are further streamlined into the three core factors (FA, FB, FC). Consequently, a four-dimensional dataset emerges and is represented as X = f(FA, FB, FC) where X = {*pp*_1_, *pp*_2_,...,*pp*_*numOfPlantPartConcepts*_}. This framework facilitates the extraction of the relation between X and f(FA, FB, FC) through the utilization of structural equations and an indicator framework. The relation can be expressed through Equation 2:


$$\begin{array}{l} F_{l}=\beta_{0}+\beta_{1}pp_{1}+\beta_{2}pp_{2}+...+\beta_{8}pp_{8}+\delta_{1}+\delta_{2}+...+\delta_{9}; \end{array}$$

(2) $$\begin{array}{l} where\text{ l}=A,B,C \end{array}$$

Additionally, the measurement models are structured across two levels:

The first level (in the previous step of Feature Reduction) for *z* = 1…, 15; *j* = 1 to 88


$$\begin{array}{l} {mp}_{1}=\lambda_{10}+\lambda_{11}AC_{z}+\;\alpha_{11}\\ :\\ mp_{j}=\lambda_{j0}+\lambda_{j1}AC_{z}+\;\alpha_{j1} \end{array}$$

The second level for *l* = A, B, C:


$$\begin{array}{l} AC_{z}=\lambda_{110}+\lambda_{111}F_{l}+\;\in_{11}\\ :\\ AC_{z}=\lambda_{j10}+\lambda_{j11}F_{l}+\;\in_{j1} \end{array}$$

These structural equations and measurement models collectively constitute the analytical framework (in Figure A.1) for the pp-mpG relation determination.

Moreover, FA, FB, and FC are then named according to their *mp*_*j*_ features having FScore_*z*_ ≥ 0.5 as follows: “beCarminative_beAntiemetic_excreteWaste_beVulnerary_beAnti Inflamation_beAntiVirus_relieveCold_relieve Pain_haveImmunity_haveDermatitisCare,” “beAntiFungi&Bac_reduceBloodlipidsBloodPressure&Bloodsugar_treatGingivitis &Scurvy_treatUrinarytract&RespiratoryDiseases_relieveSorethroat&Headache_beAntitussive,” and “reduceFever_reduceDizziness&Faint_releieveBruise_beAstringent_beExpectorant_releieveDiarrhea&Constipation_cureHemorrhoidsDisease_cureEye Disease,” respectively.

#### 3.4.2 Relation extraction (SVM technique)

There are the various *mp*_*j*_ occurrences (88 different *mp*_*j*_ features) on BVector which has 617 extracted feature vectors, and the number of different *mp*_*j*_ occurrences for the certain *pp*_*i*_ occurrence in the *h*_*k*_ document varies from 1 to 24. We then learn each *pp*_*i*_-*mp*_*j*_ pair having the pp-mp relation by SVM with supervise learning on the 617 extracted feature vectors (based on 10-fold cross validation). Moreover, there are plenty of *pp*_2_ (“*Rhizome*”) and *pp*_4_ (“*Leaf* ”) occurrences in the extracted feature vectors as 137 extracted feature vectors of *pp*_2_ and 95 extracted feature vectors of *pp*_4_. Therefore, we randomly selected 100 extracted feature vectors with *pp*_2_ or *pp*_4_ as test sample from 617 ones.

##### 3.4.2.1 pp-mp relation learning and extraction

SVM learning (Cristianini and Shawe-Taylor, 2000) with the linear kernel: The linear function, **y** = *f*(**x**), **y** is a binary vector, of the input **x** = (x_1_…x_*n*_) assigned to the positive class if *f*(**x**) ≥ 0, and otherwise to the negative class if *f*(x) < 0, can be written as follows:


$$\begin{array}{l} f\left(\text{x}\right)=\langle \text{w}\cdot \text{x}\rangle +b \end{array}$$

(3) $$\begin{array}{l} =\overset{n}{\underset{t=1}{\sum }}w_{t}x_{t}+b\; \end{array}$$

where: **x** is a dichotomous vector; **w** is a weight vector; *b* is a bias; and (w,*b*) ∈ R^*n*^ × R are the parameters that control the function.

Regarding our learning samples, SVM is applied to determine that a *pp*_*i*_ feature is related to a *mp*_*j*_ feature by the pp-mp relation. The SVM learning then determines *w*_*t*_ and *b* for *pp*_*i*_ and *mp*_*j*_ features (*x*_*t*_) in each *pp*_*i*_-*mp*_*j*_ pair with either the positive class (pp-mp_Rel, *y*_*u*_= 1, *u* = 1,2,..,9^*^88 which is 792) or the negative class (non_pp-mp_Rel, *y*_*u*_= 0) which relies on the supervised learning instances. For example (see Table A.5): the negative class of a *pp*_*i*_-*mp*_*j*_ pair occcurs if the extracted *mp*_*j*_ feature is unclear because of the noun ellipsis, e.g., the extracted *mp*_*j*_ feature is “(แก้/*relieve*)/Verb_strong_ (ปวด/*pain*)/Verb_strong_”(“*relieve pain*”) instead of “(แก้/*relieve*)/Verb_strong_ (ปวด/*pain*)/Verb_strong_ [(ท้อง/*abdomen*)/*Noun*]”(“*relieve* *abdominal* *pain*”).

Determination and extraction of the *pp*_*i*_-*mp*_*j*_ pairs having the pp-mp relation from the test sample containing *pp*_2_ or *pp*_4_ is determined by the weight vector from *pp*_*i*_ and *mp*_*j*_ features. The weight vector and the bias obtained from the SVM learning through Weka (Eibe et al., 2016) are used to test or determine and extract the *pp*_*i*_-*mp*_*j*_ pair with a pp-mp_Rel class (which is a pp-mp relation) by Equation 3. If *f*(**x**) ≥ 0, the *pp*_*i*_-*mp*_*j*_ pair with a pp-mp_Rel class (Positive-Class) as a pp-mp relation/linkage occurs, otherwise the non-pp-mp relation/linkage (Negative-Class) occurs. The *pp*_*i*_-*mp*_*j*_ pairs with Positive-Class from the test sample are collected for grouping *pp*_*i*_-*mp*_*j*_ pairs in the next step.

##### 3.4.2.2 Group *pp_*i*_*-*mp_*j*_* pairs having pp-mp relations by *pp_*i*_*

**Figure 5:**
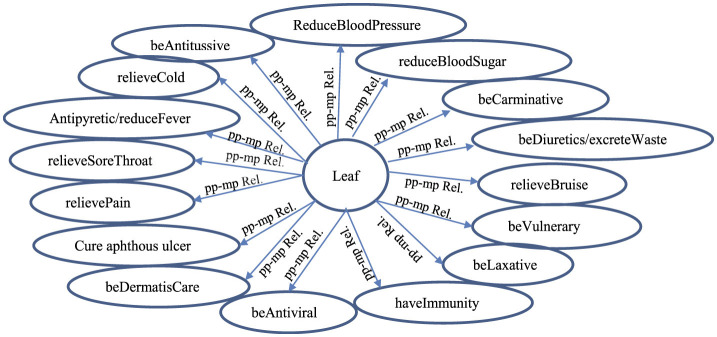
A group of *pp*_*i*_-*mp*_*j*_ pairs having pp-mp relations/linkages (pp-mp Rel) between the *mp*_*j*_ features and the *pp*_*i*_ features by SVM determination (*i* = 2; *pp*_2_ = “*Leaf* ”).

Each correct extracted *pp*_*i*_-*mp*_*j*_ pair having the pp-mp relation/linkage is collected into a group/set of *pp*_*i*_-*mp*_*j*_ pairs having the pp-mp relations with the same *pp*_*i*_ as shown in Figure 5.

## 4 Results and discussion

The 617 extracted feature vectors from 15,000 EDU corpus are employed to evaluate the proposed SEM for the pp-mpG relation extraction through the medicinal-property concept EDU identification including the *mp*_*j*_ feature extraction whilst the test sample of randomly selected 100 extracted feature vectors of pp_2_ and pp_4_ from the 617 ones are used to evaluate or test the *pp*_*i*_-*mp*_*j*_ pairs having the pp-mp relation by the SVM determination. There are four evaluations: (1) medicinal-property concept EDU identification, (2) *mp*_*j*_ feature extraction, (3) pp-mpG relation extraction by SEM and pp-mp relation determination and extraction by SVM, and (4) the evaluation of a concise and comprehensible representation of medicinal-plant property knowledge by graphical representation of pp-mpG relations.

### 4.1 Medicinal-property concept EDU identification

The medicinal-property concept EDU identification is evaluated by the percentage accuracy of the identification based on three experts with max win voting. The evaluation result of the medicinal-property concept EDU identification by SVC is 97% accurate where each medicinal-property concept is based on the VP of an EDU expression. However, there are a few medicinal-property concepts expressed within several EDU occurrences that cannot be identified by SVC, as in the following example.

EDU1: “ผู้ป่วยโควิด*-19* มีอาการปอดบวม/*A COVID-19 patient has pneumonia*”

EDU1: “หลังจาก ผู้ป่วยได้รับยาฟ้าทะลายโจร/*After the patient has received Andrographis paniculata*.”

EDU2: “[ผู้ป่วย]ก็รู้สึกดีขึ้น/[*The patient*] *feels better*.”

However, Behera and Mahalakshmi (2019) used the selected terms based on noun expressions with word size ≤ 2 from MeSH and the PubMed research articles to identify each informative sentence containing a phytochemical property feature and a target disease feature by the trained probabilistic classifier with 73% accuracy without considering the part of speech on the features since their feature positions occurred anywhere in the sentence.

### 4.2 *mp_*j*_* feature extraction

The evaluation results of extracting the *mp*_*j*_ features, which are based on the WCPattern of the medicinal-property concept EDUs after stemming words and stop word removal, by the string-matching method to wrd-CoExp ∈ word-CoExpressions of the MPC table (without the medicinal-property concept annotation) have precision of 0.91, a recall of 0.84, and an F1 score of 0.86, which are based on three experts employing max win voting. The low recall is due to the WCPattern being insufficient in covering the medicinal-property concept, i.e., the word-CoExpressions set of the MPC table is unable to match WCPattern of the following EDU.

EDU: “[ใบ] ใช้รักษาโรค มี อาการ ผิดปกติ ทางเดินอาหาร/[*A Leaf*] *is used to treat diseases with gastrointestinal disorders*.”


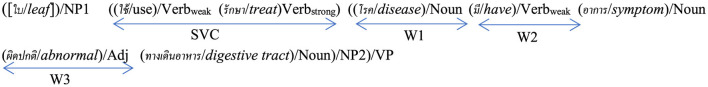


WCPattern = SVC + W1 + W2 + W3


$$\begin{array}{l} =\left(\text{ใช้}/use+\text{รักษา}/\text{treat}\right)\text{+ โรค}/\text{disease}+\text{มี}/\text{have}\\ +\text{ผิดปกติ}/\text{abnorml} \end{array}$$

However, Cho et al. (2020) used the skin-related keywords (SRKs) and data mining to extract skincare-related terms of the skincare-function categories (the medicinal properties) and medicinal herbs (at the *p*-value < 0.05) from the prescriptions. The SRKs are selected from classical texts by several experts in discussions and judgments which are time consuming and involve some subjective ideas from various references. Moreover, Jia et al. (2022) applied neural network classification as the multiclass classifier on a span-level distantly supervised named-entity recognition based on their Chinese herbal dictionary to correctly classify or predict from several classes of TCM medical named entities, e.g., symptoms, Chinese medicines, prescriptions, etc., with an F1 score of 0.77. However, the neural network classification requires the class labeling and is more complicated than using the regular dictionary and HerbMed, including the medical-symptom term list on Wikipedia, to determine our *mp*_*j*_ features without concept annotation from the documents.

### 4.3 pp-mpG relation extraction by SEM and pp-mp relation extraction by SVM

#### 4.3.1 pp-mpG relation extraction (grouped semantic-feature relation extraction)

The evaluation results of the pp-mpG relation extraction as the grouped semantic-feature relation extraction by SEM are based on the *p*-value < 0.05 or 95% accuracy with 588 pp-mpG relations correctly determined from 617 extracted binary-feature vectors from the corpus with high dimensional and correlated *mp*_*j*_ features in 50 different plant-name concepts as shown in Table 6 (by using the IBM SPSS Statistics Version 21.0 for Windows).

**Table 6:** The pp-mpG relation extraction by SEM^a^.

**pp-mpG relation**	**Estimator (coefficient)**	**S.E. (Standard Error)**	**C.R (*t*-test)**	***p*-value**
*pp*_7_ —> FA	0.163	0.097	1.682	0.093
*pp*_7_ —> FB	0.082	0.046	1.761	0.078
*pp*_6_ —> FC	−0.157	0.047	−3.342	0.001
*pp*_6_ —> FA	0.26	0.085	3.050	0.002
*pp*_6_ —> FB	0.063	0.036	1.748	0.080
*pp*_5_ —> FA	0.217	0.085	2.563	0.010
*pp*_5_ —> FB	0.078	0.044	1.773	0.076
*pp*_5_ —> FC	−0.287	0.066	−4.381	0.001
*pp*_4_ —> FA	0.219	0.072	3.040	0.002
*pp*_4_ —> FB	0.09	0.050	1.799	0.072
*pp*_4_ —> FC	−0.278	0.061	−4.566	0.001
*pp*_3_ —> FA	0.159	0.133	1.198	0.231
*pp*_3_ —> FB	0.113	0.064	1.761	0.078
*pp*_3_ —> FC	−0.169	0.068	−2.504	0.012
*pp*_2_ —> FA	0.819	0.119	6.908	0.001
*pp*_2_ —> FB	0.113	0.063	1.800	0.072
*pp*_2_ —> FC	−0.360	0.078	−4.611	0.001
*pp*_1_ —> FA	0.155	0.082	1.898	0.058
*pp*_1_ —> FB	0.099	0.055	1.796	0.072
*pp*_1_ —> FC	−0.209	0.053	−3.907	0.001
*pp*_9_ —> FA	0.864	0.256	3.372	0.001
*pp*_9_ —> FB	0.09	0.059	1.532	0.126
*pp*_9_ —> FC	−0.497	0.144	−3.460	0.001
*pp*_8_ —> FA	0.068	0.076	0.896	0.370
*pp*_8_ —> FB	0.109	0.06	1.805	0.071
*pp*_8_ —> FC	−0.215	0.053	−4.066	0.001
*pp*_7_ —> FC	−0.267	0.066	−4.029	0.001

^a^After fine-tuning the model for overall fit, it is ascertained that the harmony index stands at Chi-Square/df = 3.69, GFI = 0.935, AGFI = 0.951, RMR = 0.051, RMSEA = 0.075, all of which conform to the established criteria for a well-fitting model (Schermelleh-Engel et al., 2003; Vandenberg, 2006), where *pp1* = “*WholePlant*,” *pp*_2_ = “*Rhizome*,” *pp*_3_ = “*Bark*,” *pp*_4_ = “*Leaf and Branch*,” *pp*_5_ = “*Flower*,” *pp*_6_ = “*Fruit*,” *pp*_7_ = “*Seed*,” *pp*_8_ = “*Root*,” *pp*_9_ = “*Sucker/Shoot*.”

From Table 6, in regard to a *p*-value < 0.05, the determined pp-mpG relation with (1) the positive estimator describes increasing the number of times using *pp*_*i*_ results in increasing the improved symptom(s) by FA, FB, or FC; and (2) the negative estimator describes increasing the number of times using *pp*_*i*_ results in decreasing the symptom(s) by FA, FB, or FC. For example, (1) *pp*_2_ —> FA (with Estimator = 0.819) describes that “*increasingthenumberoftimesusingpp*_2_ *results in increasing the improved symptom(s) by FA*.” (2) *pp*_2_ —> FC (with Estimator = −0.360) describes that “*increasingthenumberoftimesusingpp*_2_ *results in decreasing the degree of symptom(s) by FC*.” However, *pp*_*i*_ – > FB cannot be determined at the *p*-value < 0.05 because most medicinal plants including indigenous medicinal plants in our corpus relate to COVID-19 remedies, which mostly exist in FA and FC. For example: leaves (of Andrographis, lemon glass, and holy basil) and rhizomes (of ginger, finger root, and galangal) that have common medicinal properties such as anti-inflammatory, antiviral, immunity and antiemetic properties conform with (Jamshidi and Cohen, 2017; Ding et al., 2019; Homnan et al., 2020; Kanjanasirirat et al., 2020; Jafarzadeh et al., 2021; Yearsley, 2021). Thus, our proposed SEM automatically extracts the grouped semantic-feature relation, especially the pp-mpG relation as the common relation which relies on 50 different herbal-name concepts and the high dimensionality of 88 *mp*_*j*_ features from the documents without involving the experts to label the relation class and to annotate the *mp*_*j*_ concepts. However, Cho et al. (2020) applied a data mining technique with skin-related keywords (SRKs), which were selected and judged by several experts, to discover medicinal herbs. SRKs were used to extract 46 medicinal herbs associated with the skincare-related terms (*p*-values < 0.05) from prescriptions of the Donguibogam text with 626 herbs without concerning the high dimensional and correlated features and also the common relation. Regarding the feature reduction, our proposed SEM to extract the pp-mpG relation reduces the *mp*_*j*_ feature dimensionality from 88 *mp*_*j*_ features to three core factors (which is reduced by 29 times) as the correlated *mp*_*j*_ feature groups (i.e., FA, FB, FC) with minimizing information loss while (Yoo et al., 2020) applied PCA (Principal Component Analysis) to reduce the protein feature dimensionality from 4,487 to 285 features (which is reduced by 16 times) as one part of all features used in deep learning to identify the medicinal uses related to the natural compounds without presenting feature correlations effect to the medicinal-use identification. According to the machine learning techniques, Pechsiri and Piriyakul (2016) applied the clustering technique to generalize the feature vector concepts as the object reduction and the feature reduction for determining a group-pair relation by Naïve Bayes from documents with 0.80 F1 score. Moreover, Braik et al. (2023) worked on the feature selection on several structural data set by the capuchin search algorithm which resulted in the information loss whilst the SEM technique for extracting the pp-mpG relation by dimensionality reduction in the *mp*_*j*_ features with minimized information loss.

**Figure 6:**
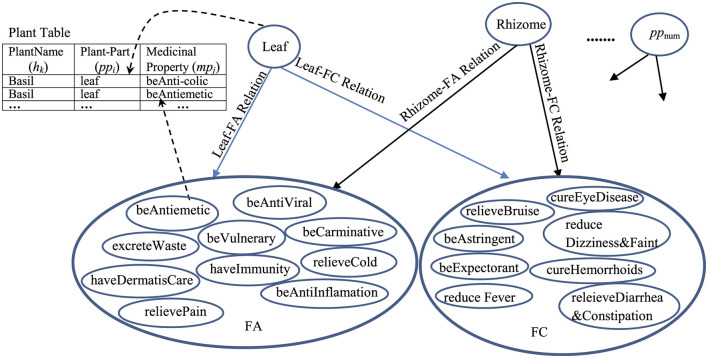
The graphical representation of the pp-mpG relations to represent the medicinal-plant property knowledge. For example: the pp-mpG relations, e.g., Leaf-FA Relation connecting a Leaf node (a *pp*4 node) to a FA node (which consists of various *mp*_*j*_ features) where the Leaf node and each *mp*_*j*_ feature in the FA node can access to the Plant table to find the certain *h*_*k*_ features as follow: (1) “Leaf” and “*Antiemetic*” are found in the following plant names: “*Basil*,” “*Andrographis paniculata*,” and “*Cinnamon*”; (2) “Leaf” and “*relieveCold*” are found in the following plant names: “*Basil*,” “*Andrographis paniculata*,” “*Fish mint*,” and “*Lemonglass*.”

The pp-mpG relations that have the *p*-value < 0.05 (Table 6) are then presented by the graphical representation to represent the medicinal-plant property knowledge (Figure 6) where Leaf-FA Relation, Leaf-FC Relation, Rhizome-FA Relation, and Rhizome-FC Relation are the pp-mpG relations/linkages, and *num* is *numOfPlantPartConcepts*. Figure 6 also includes the Plant Table collection, which is used to list the *h*_*k*_ features along with the *pp*_*i*_ and *mp*_*j*_ features.

#### 4.3.2 pp-mp relation extraction

The evaluation results of the pp-mp relation extraction by SVM with the automatic-supervised learning is evaluated in terms of an F1-score on the test sample (e.g., 350 *pp*_*i*_-*mp*_*j*_ pair instances having *pp*_2_ or *pp*_4_) based on three experts with max win voting as shown in Table 7. The correctness of extracted *pp*_*i*_-*mp*_*j*_ pairs having the pp-mp relation with an average accuracy of *pp*_2_ and *pp*_4_ is 0.80 and an average F1-score of *pp*_2_ and *pp*_4_ is 0.752, which result from having some *pp*_*i*_-*mp*_*j*_ pairs existing in both positive and negative instances of the learning example. The correct extracted *pp*_*i*_-*mp*_*j*_ pairs having the pp-mp relations also result in the correct group of *pp*_*i*_-*mp*_*j*_ pairs having pp-mp relations with the same *pp*_*i*_ (see Figure 5).

**Table 7:** Accuracies of extracting ppi-mpj pairs having pp-mp relations from the test sample.

**Plant part**	**Correctness of** ***ppi*****-*****mpj*** **pairs having pp-mp relations by**			
	**Support vector machine**			
	**Accuracy**	**Precision**	**Recall**	**F1-score**
*pp*_2_ (“*Rhizome*”)	0.79	0.763	0.728	0.745
*pp*_4_ (“*Leaf*”)	0.81	0.770	0.746	0.758

Regarding Figures 5, 6, the quantitative difference between the extracted relations/linkages by applying SEM and SVM is the number of relation/linkages connecting a certain plant-part concept to a group of different medicinal-property concepts. For example: the number of the pp-mpG relations/linkages (i.e., “Leaf-FA Relation” and “Leaf-FC Relation” (Figure 6) connect “Leaf” to FA and FC, respectively) determined and extracted by SEM is two linkages whereas the number of pp-mp relations/linkages (i.e., each “pp-mp Rel” linkage (Figure 5) connects “Leaf” to different *mp*_*j*_ features) extracted by SVM is 16 linkages. This results in the time-consuming extraction the relations/linkages. The qualitative difference between the extracted relations/linkages by SEM and SVM is that the pp-mpG relations/linkages extracted by SEM have the correlated *mp*_*j*_ feature occurrences within the medicinal-property concept group (e.g., FA, FC in Figure 6) whereas there is no correlation occurrence among the *mp*_*j*_ features in a group of extracted pp-mp relations/linkages with the same *pp*_*i*_ feature by SVM as shown in Figure 5. Other than SVM, using the neural network technique as deep learning to extract the medicinal uses in diseases related to the natural compounds is inadequate (Yoo et al., 2020) as it cannot present the feature-correlation occurrences among their independent variables/features (e.g., chemical property features in the natural compounds) of the extracted relations.

However, the extracted pp-mpG relations by SEM result in the synergistic effects when using different *pp*_*i*_ features, e.g., using “Leaf” and “Rhizome” together for cold relief as shown in Figure 6 where both “Leaf-FA Relation” and “Rhizome-FA Relation” connect to FA containing the correlated *mp*_*j*_ features “beAntiViral” and “relieveCold.” Thus, Figure 6 provides the medicinal-plant property knowledge for potential usages in the alternative medical therapy for synergistically solving the general health problems/symptoms.

In summary, the fundamental principle for proving the relationship linkage between SEM and SVM methodologies demonstrates that when there are n plant components and k medicinal-plant properties, the relationship testing requires n^*^k connections, whereas if we reduce the dimension k to only m = k/c (where c > 0), this results in the linkage analysis requiring only n^*^m connections (where n^*^m < n^*^k). This dimensional reduction approach significantly decreases computational complexity by reducing the total number of relationship pathways that need to be analyzed, thereby improving processing efficiency while maintaining the integrity of the plant component-medicinal property relationship modeling framework through the integrated SEM-SVM methodology.

### 4.4 Evaluation of concise and comprehensible representation of medicinal-plant property knowledge by graphical representation of pp-mpG relations

**Figure 7:**
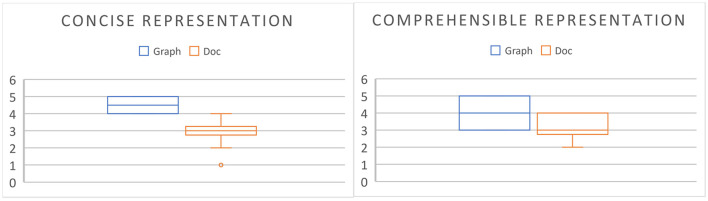
Box plot of the concise and comprehensible representations by Doc and Graph with the Likert scale 1–5.

We evaluate the concise and comprehensible representations of the medicinal-plant property knowledge by the graphical representation of the pp-mpG relations in terms of a Likert scale (1–5). The evaluation results with the average scores based on the Likert scale of the concise and comprehensible representations of Doc (which is the documents from the pharmacy academic websites) and Graph (which is the graphical representation of the pp-mpG relations) by the 30 end-users (who are non-professional persons) are presented in Figure 7, where the average scores of the concise representation by Graph and Doc are 4.5 and 2.9, respectively, and where the average scores of the comprehensible representation by Graph and Doc are 4.0 and 3.1, respectively.

Therefore, the contribution of our research, particularly Figure 6, can enhance the ability of non-professional persons in exploiting the potential of using herbal medicines or medicinal plant property knowledge as an alternative medical therapy through social media.

**Figure 8:**
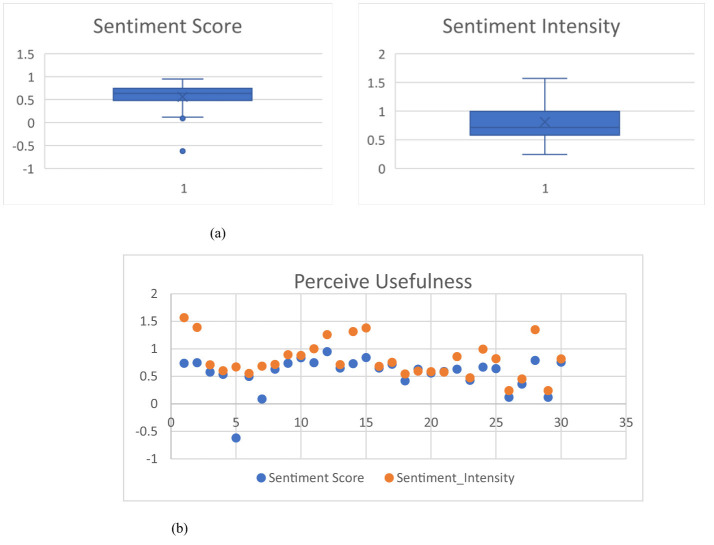
The sentiment graphs of 30 user reviews. **(a)** Box plot of sentiment score and sentiment intensity. **(b)** Scatter plot between sentiment score and sentiment intensity.

Moreover, we also collect the reviews/comments on the community forums (https://community.breastcancer.org/en/discussion/827489/turmeric-curcumin-yes-or-no; accessed on 30 August 2024; https://www.mtbr.com/threads/turmeric-im-a-believer.1202573/; accessed on 30 August 2024) of general users about the usefulness of using a certain medicinal plant, e.g., turmeric/curcumin as an alternative medical therapy to solve their health problems according to the various information on a particular medicinal plant on social media. In relation to the review/comment collection, we randomly select 30 reviews/comments from different users in using turmeric/curcumin as an alternative medical therapy for both cancer pains and inflammation or joint/muscle pains, to determine a sentiment score, a magnitude value, and a sentiment intensity (Lamba and Madhusudhan, 2022; Rutkowska and Szyszko, 2024) of each user review (30 user reviews/comments) by using text2data (https://www.text2data.com) as shown in Table A.4. The text2data software is a dictionary-based analyzer. The sentiment score is a numerical value ranging from −1.0 (extremely negative) to +1.0 (extremely positive), reflecting the overall emotional polarity expressed in a given text, indicating whether the expressed opinion is generally negative, neutral, or positive. In contrast, the sentiment magnitude measures the strength or intensity of the sentiment, regardless of polarity; it is a non-negative value that increases with the emotional expressiveness of the content, with higher magnitude values indicating more emotionally charged language (Lin et al., 2020). We also present a sentiment score graph and a sentiment intensity graph with the averages of the sentiment score and the sentiment intensity as 0.56, and 0.81, respectively, as shown in Figure 8a and a perceive usefulness graph between the sentiment scores and the sentiment intensity values as shown in Figure 8b.

Figure 8b shows that most of the scatter plots are higher than 0.5 which means “Usefulness” or “Satisfaction” on using the medicinal-plant properties as a herbal medicine through the information on the networks, where a positive score means “Usefulnesss,” a zero score means neutral, and a negative score means “Dissatisfaction.” Thus, the medicinal-plant property knowledge representation in Figure 6 through social media can potentially enhance positive Usefulness to be the higher than the current one.

### 4.5 Limitations of study

This study explores the potential use of SEM as a statistical approach and SVM as the machine learning technique to extract the semantic relation from the corpus of the downloaded documents on the Thai pharmacy academic websites as the sources of data. The limitations of our research rely on the corpus behavior study which remains crucial for selecting the most effective techniques to address a research gap because different domains (like news, academic writing, fiction, etc.) have distinct patterns of language use. A word's frequency, collocations, grammatical patterns, and even its overall significance can all change when moving from one domain to another. For example, according to the Thai-herbal-plant corpus of the herbal domain, the medicinal-property concept feature (*mp*_*j*_) based on WCPattern within five words on an EDU verb phrase. In contrast to the plant-disease corpus of the agriculture domain, a plant-disease symptom concept is represented by an effect verb concept of an EDU's verb phrase (Pechsiri and Piriyakul, 2010), e.g., EDU: “ใบหงิก/*Leaves shrink*” (“(((ใบ/*leaves*)/noun)/NP ((หงิก/*shrink*)/verb)/VP)/EDU”) where (หงิก/*shrink*)/verb is the plant-symptom concept. In addition, a human symptom concept in the healthcare domain requires at least a 2-word cooccurrence to represent the symptom concept, e.g., EDU: “[ผู้ป่วย]ปวดศรีษะ/*A patient feels headache*” (“([((ผู้ป่วย/*patient*)/noun)/NP] ((ปวด/*pain*)/verb (ศรีษะ/head)/noun)VP)/EDU”) where (ปวด/*pain*)/verb+(ศรีษะ/head)/noun is a 2-word cooccurrence (verb+noun) having a symptom concept; Pechsiri and Piriyakul, 2016). Moreover, our herbal data source is provided by academic pharmacy organizations to ensure the accuracy of the information. Therefore, the corpus behaviors can influence the performance for use of statistical approach and also machine learning approach. Further exploration also needs to be made on the use of such approaches in other diagnostic cases [e.g., LCA study (Pechsiri et al., 2025)] to evaluate the potential use for other real-world applications.

## 5 Conclusion

This research applied word-co expression as the compound variable based on WCPattern (which is the verb-based word-co pattern) to determine the *mp*_*j*_ features to extract the grouped semantic-feature relations (the pp-mpG relations) without the relational-class labeling and the pp-mp relations on supervised learning from the downloaded documents on the pharmacy academic websites. The limitations of our *mp*_*j*_ feature extraction are (1) the *mp*_*j*_ feature needs to be expressed within a single EDU occurrence, and (2) the medicinal-property concept feature needs to be expressed within five words on an EDU verb phrase. The extraction of the pp-mpG relations and the pp-mp relations from the documents relies on (1) the medicinal-property concept EDU identification by SVC collected from the verb-phrases of the EDU_tmt_ expressions from HerbMed and Wikipedia; (2) the *mp*_*j*_ feature extraction (without annotating concepts) by using the MPC table based on WCPattern to HerbMed and Wikipedia, and (3) the pp-mpG relation extraction by SEM involving the feature correlation among dependent variables (i.e., the *mp*_*j*_ features) with high dimensionality. The result of each extracted pp-mpG relation as a linkage connecting a *pp*_*i*_ feature node to the certain *mpG*_*g*_ nodes from the documents has become the graphical representation of the medicinal-plant property knowledge. The relation/linkage qualities, e.g., the number of linkages (which affect the time it takes to extract the relations/linkages) and the correlated *mp*_*j*_ feature expressions (which result in remedy enhancement with the supplementary treatment), of the pp-mpG relations by the proposed SEM technique are superior to the pp-mp relations extracted by SVM with supervised learning. However, the dominant *mp*_*j*_ feature of *mpG*_*g*_ in each disease remedy should be determined in future research. Thus, the graphical representation of the pp-mpG relations including Plant Table (Figure 6) provides concise and comprehensible representations of the medicinal-plant property knowledge for the potential enhancement of usefulness in alternative medical therapy through social media. Furthermore, the alternative medicinal usages can benefit users in terms of low cost, fewer side effects or a complete lack of side effects, and synergistic effects (Behera and Mahalakshmi, 2019). Finally, the grouped medicinal-plant property relation can be applied to several languages, particularly in East and Southeast Asia such as Tibetan, Loa, Japanese, and Chinese, having the similar behaviors of the Thai language, e.g., noun phrase ellipsis and lack of a sentence or word delimiter which affect computational tasks in natural language processing to handle these textual data effectively. Furthermore, integrating the grouped medicinal-plant property relation into the healthcare system establishes an enhanced problem-solving framework. Moreover, our grouped semantic-feature relation method can be applied not only to the grouped medicinal-plant property relation, but also to the other business areas, e.g., customer's behavior relates to various groups of product attributes on the social medias. The customer behavior is then influenced by the social medias containing various groups of product attributes. Finally, these attributes shape how consumers evaluate, choose, and ultimately purchase products.

## Data Availability

The original contributions presented in the study are included in the article/Supplementary material, further inquiries can be directed to the corresponding author. Plant Genetic Conservation Project under the royal initiative of Her Highness Princes Maha Chakri Sirindhorn (http://www.rspg.or.th/), Medicinal Plant Information by Faculty of Pharmacy at Silpakorn University (https://pharmacy.su.ac.th/herbmed/herb/text/), Herbal Information Center in Faculty of Pharmacy Mahidol University (https://medplant.mahidol.ac.th/document/inews.asp), and the community forums (https://community.breastcancer.org/en/discussion/827489/turmeric-curcumin-yes-orno; https://www.mtbr.com/threads/turmeric-im-a-believer.1202573/).
