# Supplementary material for: Grouped semantic-feature relation extraction from texts to represent medicinal-plant property knowledge on social media
Source: Front Artif Intell. 2025 Aug 8;8:1579357. doi: 10.3389/frai.2025.1579357 (PMC12371932; doi:10.3389/frai.2025.1579357)
Supplement: Supplementary file 1 [file Table_1.docx]

| Literatures | Corpus or Data set | Research Goal | Technique | Evaluation |
| --- | --- | --- | --- | --- |
| Fang et al. [14] 2008 | PubMed literature corpus; Annotation | Association: (TCM, disease); ( TCM, ingredient) | Rule-based information extraction | Average precision:0.91 |
| Choi and Lee [15] 2015 | PubMed abstracts | Herb-chemical relationships | Rule-based text mining model | F-measure: 0.749 |
| Behera and Mahalakshmi [16] 2019 | biomedical literatures on PubMed | Disease based upon the phytochemical properties of medicinal plants | Text mining techniques including a trained probabilistic classifier | 73% Accuracy |
| Cho et al. [17] 2020 | Donguibogam texts in medicinal herbs for skincare | Associations between medicinal herbs and skincare-related functions based on 26 skin-related keywords (SRKs) | Data mining technique | 46 Herbs out of 52 candidate medicinal herbs had skincare-related effects |
| Yoo et al. [18] 2020 | Structured data (latent knowledge features) and unstructured data (molecular interaction features) | Identifying the medicinal uses in diseases related to the natural compounds | Principal Component Analysis (PCA) to reduce the protein feature dimensionality from 4,487 to 285 features   - **Feature reduction** | an average AUROC value in the relation: 0.9 |
| Zhang et al. [19] 2020 | Traditional Chinese Medicine (TCM) book | Prediction/classification of the named-entity type on a multiclass model and the named-entity boundary detection | Neural network learning | An average F1 scores of 0.7 |
| Jia et al. [20] 2022 | TCM book | Determining named entities, e.g., Chinese medicine, with manual class labeling | Simple multilayer neural network classifier for multiclass classification | F1 score of 0.77 |
| Pechsiri and Piriyakul [21] 2016 | Hospital-web-board documents | Automatically extracting a semantic relation between two event-explanation groups as a Problem-Solving relation / DiseaseSymptom-Treatment relation | Object clustering and Feature clustering for reducing the object and feature dimensions before learning the relation by Naïve Bayes  - **Object/Feature reduction** | F1score of 0.81 |
| Braik et al. [22] 2023 | Data set from https://jundongl.github.io/scikit-feature/datasets.html. | Feature Selection to lower the feature dimensionality for classification by machine learning | Capuchin Search Algorithm (CSA)  **-Feature reduction** | on the COVID-19 datasets with an average accuracy of 95.9% in classification after applying CSA with the low fitness value of 0.04 |

Table A.1 Related Work Summary
