# Supplementary material for: Grouped semantic-feature relation extraction from texts to represent medicinal-plant property knowledge on social media
Source: Front Artif Intell. 2025 Aug 8;8:1579357. doi: 10.3389/frai.2025.1579357 (PMC12371932; doi:10.3389/frai.2025.1579357)
Supplement: Supplementary file 4 [file Table_4.docx]

Table A.4 Show Sentiment Score, Magnitude Value, and Sentiment Intensity from 30 user reviews/comments

| Review | Doc_length (words) | Sentiment Score | Magnitude Value | Sentiment_Intensity |
| --- | --- | --- | --- | --- |
| 1 | 62 | 0.74 | 3.50 | 1.568 |
| 2 | 60 | 0.75 | 2.89 | 1.392 |
| 3 | 20 | 0.58 | 1.01 | 0.709 |
| 4 | 93 | 0.536 | 0.760 | 0.6032 |
| 5 | 84 | -0.62 | 3.69 | 0.673 |
| 6 | 12 | 0.50 | 0.68 | 0.554 |
| 7 | 70 | 0.09 | 2.08 | 0.687 |
| 8 | 51 | 0.63 | 0.92 | 0.717 |
| 9 | 56 | 0.74 | 1.25 | 0.893 |
| 10 | 40 | 0.84 | 0.98 | 0.882 |
| 11 | 63 | 0.75 | 1.59 | 1.002 |
| 12 | 75 | 0.95 | 1.98 | 1.259 |
| 13 | 109 | 0.65 | 0.86 | 0.713 |
| 14 | 11 | 0.73 | 2.68 | 1.315 |
| 15 | 130 | 0.843 | 2.63 | 1.3791 |
| 16 | 53 | 0.65 | 0.76 | 0.683 |
| 17 | 29 | 0.72 | 0.835 | 0.7545 |
| 18 | 25 | 0.42 | 0.83 | 0.543 |
| 19 | 12 | 0.63 | 0.52 | 0.597 |
| 20 | 50 | 0.56 | 0.64 | 0.584 |
| 21 | 24 | 0.59 | 0.55 | 0.578 |
| 22 | 108 | 0.632 | 1.39 | 0.8594 |
| 23 | 17 | 0.43 | 0.58 | 0.475 |
| 24 | 177 | 0.67 | 01.75 | 0.994 |
| 25 | 131 | 0.64 | 1.25 | 0.823 |
| 26 | 37 | 0.12 | 0.53 | 0.243 |
| 27 | 36 | 0.36 | 0.67 | 0.453 |
| 28 | 132 | 0.79 | 2.65 | 1.348 |
| 29 | 20 | 0.12 | 0.53 | 0.243 |
| 30 | 7 | 0.76 | 0.954 | 0.8182 |
