# Supplementary figures and images for: Grouped semantic-feature relation extraction from texts to represent medicinal-plant property knowledge on social media
Source: Front Artif Intell. 2025 Aug 8;8:1579357. doi: 10.3389/frai.2025.1579357 (PMC12371932; doi:10.3389/frai.2025.1579357)

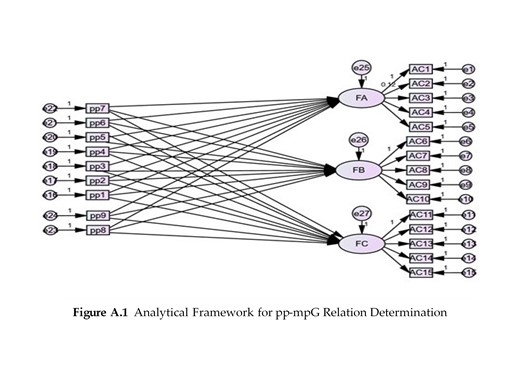

Supplement: Supplementary file 6 [file Image_1.jpeg]
